# A ternary eutectic solvent for cellulose nanocrystal production: exploring the recyclability and pre-pilot scale-up

**DOI:** 10.3389/fchem.2023.1233889

**Published:** 2023-08-25

**Authors:** Mayra A. Mariño, Maria G. Paredes, Natalia Martinez, Daniela Millan, Ricardo A. Tapia, Domingo Ruiz, Mauricio Isaacs, Paulina Pavez

**Affiliations:** ^1^ Department of Chemical Engineering, Universidad de Concepción, Concepción, Chile; ^2^ Facultad de Química y de Farmacia, Pontificia Universidad Católica de Chile, Santiago, Chile; ^3^ Departamento de Química, Universidad Técnica Federico Santa María, San Joaquín, Chile; ^4^ Centro Integrativo de Biologia y Quimica Aplicada (CIBQA), Universidad Bernardo O’Higgins, Santiago, Chile; ^5^ Laboratorio de Materiales Electrocerámicos, Facultad de Química y Biología, Universidad de Santiago de Chile, Santiago, Chile; ^6^ Centro de Nanotecnología y Materiales Avanzados (CIEN-UC), Pontificia Universidad Católica de Chile, Santiago, Chile; ^7^ Millennium Institute on Green Ammonia as Energy Vector Avda Vicuña Mackenna, Santiago, Chile

**Keywords:** deep eutectic solvents, ternary solvents, recyclable media, nanowhiskers, cellulose

## Abstract

Deep eutectic solvents (DES) formed using choline chloride (ChCl), *p*-toluenesulfonic acid (*p*TSA) of stoichiometry ChCl: pTSA (1:1) and (1:2), and its ternary eutectic mixtures with phosphoric acid (PA) 85% as an additive (ChCl: *p*TSA: PA) were evaluated for cellulose nanocrystal (CNC) isolation. Initially, the hydrolytic efficiency to produce CNC of each DES was compared before and after adding phosphoric acid by Hammett acidity parameters and the Gutmann acceptor number. Moreover, different DES molar ratios and reaction time were studied at 80°C for CNC optimization. The nanomaterial characteristics were analyzed by field emission scanning electron microscopy (FESEM), X-ray diffraction (XRD), Fourier-transform infrared spectroscopy (FTIR), and thermogravimetric analysis (TGA). The ternary eutectic mixture ChCl: *p*TSA: PA molar ratio (1:1:1.35) was chosen as a suitable recyclable ternary system at the laboratory scale. A CNC yield of about 80% was obtained from the hydrolysis of commercial cellulose in five cycles of recovery, but it dropped to 35% in pre-pilot scaling. However, no variation in the average size of the resulting CNC was observed (132 ± 50 nm x 23 ± 4 nm), which presented high thermal stability (Tmax 362°C) and high crystallinity of about 80% after 3 h of reaction time.

## 1 Introduction

Cellulose, the most abundant renewable polymer resource available today, is considered a rational raw material to produce green and biocompatible products. This natural polymer consists of repetitive D-glucose units linked through *β*-(1,4)-glycosidic bonds and has unique structural and physical properties. The properties of plant cellulose result from multiple hydrogen-bonding interactions, generating a semi-crystalline polymer with crystalline and amorphous regions, although there are two other sources, bacterial and marine sources from tunicates, which are morphologically different (Yu et al., 2021). Recently, the use of cellulose in the preparation of cellulose nanocrystals (CNC) has attracted attention due to its outstanding structural characteristics, such as excellent mechanical strength, high stiffness, and potential chemical functionality. CNC are usually obtained by acidic hydrolysis of cellulose-based starting materials, using mineral acids like sulfuric, hydrochloric, hydrobromic, and phosphoric acids (Trache et al., 2020). Hydrolysis with sulfuric acid is a widely used method and has been optimized (H_2_SO_4_ 64 wt% at 45 °C for 30–60 min) to obtain moderate CNC yields (20%–30%) but with the lower thermal stability of the nanoparticles (180 °C–220 °C) due to sulfate groups generated by esterification of surface hydroxyl groups (Roman and Winter, 2004). In addition, CNC obtained using phosphoric acid has gained interest because the nanomaterial obtained has greater thermal stability (Vanderfleet et al., 2018; Frost and Johan Foster, 2020). In addition, both inorganic esterification products resulted in negatively charged stable suspensions in polar solvents. However, a large volume of waste effluents is generated using inorganic acids and byproducts obtained via amorphous cellulose over-degradation (Raza and Abu-Jdayil, 2022), which led to the research of a recyclable media using organic acids, such as oxalic acid, maleic acid, and *p*-toluenesulfonic acid (*p*TSA). These organic acids could be recovered by recrystallization once the corresponding esterification and hydrolysis products (oxalate, maleate, and sulfate CNC, respectively) are previously isolated by filtration. Additionally, the milder acidity of these organic acids produced high CNC yields (40%–80%) along with lower degradation byproducts but required long reaction times (4–7 h) (Li et al., 2017; Bian et al., 2019; Wang et al., 2019; Jiang et al., 2021).

Recently, other emerging alternatives include deep eutectic solvents (DES), a form of liquid recyclable media due to their chemical stability and low vapor pressure. DES are organic liquids obtained by the interaction of a hydrogen bond acceptor (HBA), which is usually a halide quaternary ammonium salt, and a hydrogen bond donor (HBD), such as alcohols, amines, or carboxylic acids, among others. DES act as isolation media or reaction media like ionic liquids (ILs) but also present additional advantages. These solvents provide ease of preparation (sometimes without post-synthesis purification) and lower toxicity since their precursors are usually plant primary metabolites (sugar, alcohols, amino acids, terpenes, amines, etc.) (Ma et al., 2019; Raza and Abu-Jdayil, 2022).

If the mixture that forms the eutectic solvent is made up of more than one natural component, it is called a natural deep eutectic solvent (NADES). NADES can be prepared with molar ratios of two or more hydrogen bond donors so that they are tunable solvents and can also act as recyclable catalysts in organic and enzymatic reactions (Liu et al., 2018). These remarkable properties of green eutectic solvents have led to the development of various technologies, especially for the solubilization of plant biomolecules with potential applications in the food, cosmetic, and pharmaceutical industries, such as natural colorants, flavonoids, coumarins, spirulina, essential oils, alkaloids, and steroids, among others (da Silva et al., 2021; Saini et al., 2022).

Another attractive application of NADES is the lignocellulose component separation for the valorization of agro-industrial waste, known as biorefining. In this context, high yields and selectivity for lignin and cellulose fractionation have been achieved by the study of the NADES’ polarity. Recyclable extraction media using acidic NADES have been efficient in providing CNC and cellulose nanofibers (CNF) by selective hydrolysis of amorphous cellulose (Bjelic et al., 2022). Large volumes of NADES have been used to obtain CNC/CNF by simple stirring and heating of its organic components assisted by ultrasound; among them, those that stand out are choline chloride: oxalic acid dihydrate (Sirviö et al., 2016; Yu et al., 2020; Douard et al., 2021), choline chloride: urea, choline chloride: glycerol (Lim et al., 2022), betaine: oxalic acid dihydrate (Deng et al., 2022), choline chloride: formic acid (Wu et al., 2021), choline chloride: *p*TSA, choline chloride: levulinic acid (Sirviö et al., 2016), and choline chloride: lactic acid (Liu et al., 2019). The synthesis of CNC using binary eutectic mixtures usually requires reaction times of 3–6 h at temperatures between 60 °C and 120 °C and ultrasound treatment of 20–30 min. DES choline chloride: oxalic acid dihydrate (ChCl/OAD) is the most studied, and CNC is obtained in a 55% yield from bleached rice straw treated at 80 °C for 4 h (Lim et al., 2022) and in a 35% yield from cotton fiber at 95 °C for 6 h (Douard et al., 2021).

Ternary mixtures have been used to speed up the process of biomass deconstruction and optimization of the size on the nanometric scale of lignin-containing cellulose nanofibers (LCNF) according to the acidity of the eutectic medium. Ternary carboxylic acid deep eutectic solvents (TCADES) like ChCl :OA: AlCl_3_.6H_2_O (1:1:0.2) (Ji et al., 2021), ChCl: lactic acid: *p*TSA (2:10:1) (Shu et al., 2021), and ChCl: OA: *p*TSA (2:1:1) (Jiang et al., 2021) were reported for fractionation of sugarcane bagasse treated for 20 min at 100 °C (microwave-assisted plus 30 min ultrasound-assisted), poplar treated for 3 h at 100 °C (blender-assisted for 3 min plus micro-fluidization), and softwood pulp treated for 3 h at 80 °C (blender-assisted for 30 min), respectively. In contrast, only one study described the effectiveness of ternary eutectic mixtures to obtain CNC using ChCl: OAD with FeCl_3_.6H_2_O as an additive (molar ratio 4.43:1:0.15). Hydrolysis of bleached eucalyptus kraft pulp at 80 °C for 6 h yielded 73% of CNC (length 270 ± 92). However, recovery of this ternary system involves a four-step process (Yang et al., 2019).

In this work, we propose a simple methodology to obtain CNC from CFII commercial cellulose using two choline chloride/*p*-toluenesulfonic (ChCl: *pTSA*) acid deep eutectic solvents of different stoichiometry DES 1:1 and DES 1:2, Figure 1 and ternary eutectic mixtures (ChCl: *pTSA*: PA) adding phosphoric acid (PA) to form (ChCl: *pTSA*: PA) of different stoichiometry. The addition of phosphoric acid (PA) aims to improve the thermal stability of the CNC obtained. In this context, binary and ternary acidic conditions according to different molar ratios of ChCl: *p*TSA and PA will be used as an acid catalyst and a solvent, respectively, to obtain CNC at 80 °C. Moreover, the physicochemical properties and yields of CNC resulting from different hydrolysis conditions were explored (X-ray diffraction, thermogravimetric analysis, field emission scanning electron micrscopy, and scanning electron microscopy), as well as the recycling cycles. The efficiency of the process (CNC performance) and the characteristics of the nanomaterial obtained were evaluated based on the structure of DES, the amount of phosphoric acid added, and the acidity of the solvent measured by the Gutmann acceptor number (AN) using a ferrocifen probe and in terms of Hammett acidity parameters (*H*_0_) using 4-nitroaniline as an indicator.

**FIGURE 1:**
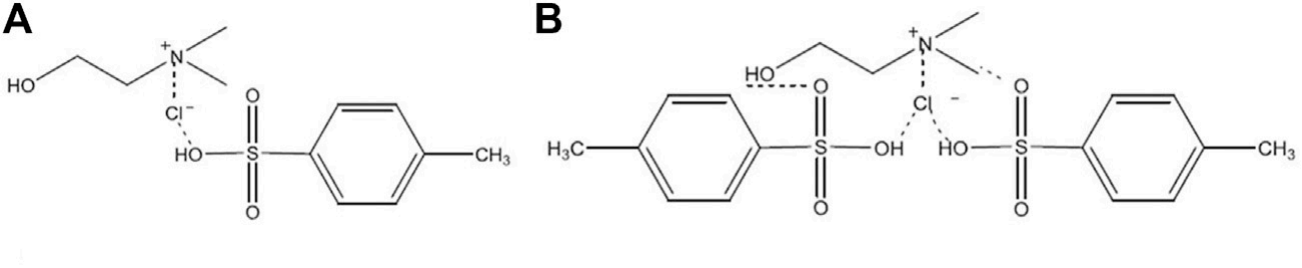
Binary eutectic mixture **(A)** ChCl: *p*TSA (DES1:1) and **(B)** ChCl: *p*TSA (DES1:2).

## 2 Experimental section

Materials: the probe ferrocifen was purchased from BOC Sciences and used without further purification. Choline chloride (≥98%), *p*-toluenesulfonic acid (≥98%), 4-nitroaniline (%), cellulose powder (CFII), and activated charcoal were purchased from Sigma-Aldrich.

### 2.1 Synthesis of DES

The DES was synthesized according to the literature in a round-bottom flask. Choline chloride and *p*-toluenesulfonic acid at the desired molar ratio were added, followed by magnetic agitation for 4 h at 60 °C (Assanosi et al., 2014). The chemical structure of the yellow pale liquids resulting herein, assigned as DES 1:1 and 1:2, was confirmed by ^1^H NMR, ^13^C NMR, FT-IR, and Raman spectroscopy (see Supplementary Figures S1–S4). Ternary mixtures were prepared, following Rodriguez Rodriguez et al. (2019) and Wang et al. (2022).

### 2.2 Determination of the Hammett acidity function (H_0_) of DES ChCl: pTSA (1:1), (1:2), and ternary eutectic mixtures (ChCl: pTSA: PA)

The acidity of each binary and ternary DES mixture in terms of *H*_0_ was measured by UV-visible spectroscopy, according to a reported methodology (Kore et al., 2012; Suzuki et al., 2018), using 4-nitroaniline as an indicator in water. The *H*_0_ value is calculated by Eq. 1, where (I) is the absorbance of the unprotonated form of the indicator and (IH^+^) is the absorbance of the unprotonated form of 4-nitroaniline as Eq. 1.

$$H_{0}=pK{\left(I\right)}_{aq}+\log\left[\frac{\left(I\right)}{\left({IH}^{+}\right)}\right].$$ (1)

The *H*_0_ values were determined under the following experimental conditions: concentration of 4-nitroaniline (3 mgL^-1^, p*K*(I)_aq_ = p*K*_a_ = 0.99) and concentration of each DES (50 mmol·L^-1^) in aqueous solution. The maximal absorbance of the unprotonated form of the indicator is observed at 378 nm in water. Supplementary Figure S5 shows the decrease in the absorbance in the unprotonated form of the indicator (I) in aqueous solutions after the addition of each DES. The *H*_0_ value would indicate the relative proton donation capacity of each DES in water.

### 2.3 Determination of the Gutmann number (AN) of DES ChCl: pTSA (1:1), (1:2), and ternary eutectic mixtures (ChCl: pTSA: PA)

A stock of ferrocyphen dye was prepared in a concentration of 8.10^−3^ M. Then, 10 µL of the dye was dissolved in 600 µL of each DES. Previously, the probe was homogenized and stabilized for 24 h. The measurements were performed in a diode array spectrophotometer HP 8453 at 25 °C ± 0.1 °C using a spectral software application. See Supplementary Figure S6.

### 2.4 Preparation of CNC by acid hydrolysis using DES ChCl: pTSA (1:1), (1:2), and ternary eutectic systems (ChCl: pTSA: PA)

Initially, cellulose (3% w/w based on the mass of DES) hydrolysis was studied with each DES at 80 °C in an oil bath under constant stirring at 500 rpm for 1–3 h. The amount of cellulose used for each experiment was based on the experimental conditions shown in Abushammala et al. (2015) and Lazko et al. (2016). Once hydrolysis finished, 20 mL of deionized cold water was added and centrifuged for 15 min at 9,600 rpm in a centrifuge (5804 Eppendorf AG, Barkhausenweg). This washing procedure was repeated three times. The resulting supernatant was sonicated (12 min) (Sonifier 450, Branson Ultrasonics) at 40% of the nominal amplitude. Then, the solution was centrifuged for 10 min at 4,600 rpm. The supernatant was oven-dried at 60°C for 3 h. Additionally, to establish the effect of phosphoric acid on the hydrolysis reaction, the preparation of CNC was also tested using the same previous procedure but using phosphoric acid at 10% and 40% wt (at 80 °C for 3 h). The results are shown in Supplementary Table S1. Each experiment was performed in triplicate using a mass ratio of 30:1 for DES to cellulose. The final yield of CNC was calculated using Eq. 2:

$$Yield\;CNC\;\left(\%\right)=\frac{Mass\;of\;CNC\;obtained}{Mass\;of\;cellulose}*100.$$ (2)

### 2.5 FESEM and STEM analysis

Morphological features and the average length and width of CNC obtained in each experimental condition were investigated using a Quanta field emission scanning electron microscope 250 FESEM (FEI, Quanta™) or scanning transmission electron microscope (STEM) techniques. The sample for FESEM was obtained by dispersing 48 mg of solid nanocellulose in 5 mL of water. This sample was sonicated for 12 min. Then, a 100-μL aliquot was diluted in 900 µL of water and placed on a silicon plate (Si wafer (100) 4″ × 0.525 mm, N-type P doped) and left to dry at room temperature. This sample was covered with gold by sputtering using a MED 020 Sputter (BalTec, Balzers, Liechtenstein), and then analyzed in the electron microscope. The sample for STEM analysis was prepared according to Reyes et al. (2019) and Wang et al. (2019). Briefly, a 50-µL aliquot of dispersed nanocellulose (48 mg/5 mL) was diluted in 10 mL of water; then, the sample was placed on a copper grid without further treatment. Histograms were made using ImageJ software, which is freely available. Each micrograph was analyzed by marking the width and length of each particle, where at least 40 were measured to collect data with a statistical value. For FESEM images and histograms from dimensional analysis for all CNC obtained in this study, including both FESEM and histogram images of CNC obtained in the last recycled step and CNC obtained in the reactor scale, see Supplementary Figure S7.

### 2.6 Thermogravimetric analysis

Thermogravimetric analysis (TGA) was performed on a TGA 4000 (Perkin Elmer) system with an autosampler, 100–240V, 50/60 Hz. The heating temperature started from 30 °C to 800 °C at 20 °C/min under a nitrogen gas flow of 20 mL/min. Approximately 5 mg of dried CNC was used for the analysis (Supplementary Table S2).

### 2.7 X-ray diffraction analysis

X-ray diffraction analysis (XRD) data were collected with a Bruker D2 PHASER diffractometer and Cu_Kα_ radiation *λ* = 1.5406 Å at 30 kV and a 10 mA power source. Diffractograms were collected in a 2θ range of 5°–80° at the rate of 0.02°/s. The crystallinity index (CrI) from powder X-ray diffraction data was calculated by the subtraction method using Eq. 3:

$$CrI=\frac{Area\;of\;crystalline\;peaks}{Area\;of\;all\;peaks\;\left(crystalline+amorphous\right)}*100.$$ (3)

The Scherrer equation (Kang et al., 2018) was used to calculate the crystal size L (nm) of the cellulose structure in respect of the (200) plane (Eq. 4):

$$L\;\left(h,k,l\right)=\frac{k\lambda }{bcos\theta }.$$ (4)

Here, k is the correction factor and is usually considered 0.91, λ is the radiation wavelength, θ is the diffraction angle, and b is the corrected angular width (in radians) at half maximum intensity.

### 2.8 Fourier-transform infrared spectroscopy (FTIR)

The changes in the chemical structure of the CNC obtained in this work were determined by Fourier-transform infrared spectroscopy (FTIR). The samples (2 mg) were prepared in 200 mg of KBr and pressed into a transparent pill before measurements. The spectra were recorded on an FTIR iS10 FTIR-ATR spectrophotometer in a transmittance range of 4,000–450 cm^-1^. See Supplementary Figure S8 for the FTIR experiment.

### 2.9 Recyclability analysis

Specifically, the ternary eutectic mixture ChCl: *p*TSA: PA (1:1:1.35) was recycled and reused (no need to add more phosphoric acid, PA) under our reaction conditions to obtain CNC, which was 3 h of hydrolysis at 80 °C. ChCl: *p*TSA: PA (1:1:1.35) was recovered from residual water collected after the hydrolysis and purification of the CNC obtained. To remove the sugar in the recycled ChCl: *p*TSA: PA, this DES was dissolved in anhydrous dichloro-methane (100 mL) and stirred overnight at 50 °C in the presence of active charcoal (1% in weight) (Park et al., 2016). Then, a black solution was filtered, obtaining a decolorized mixture of water–ChCl: *p*TSA: PA, which was vacuum distilled at 55 °C for 4 h to remove most of the water and concentrate the ternary eutectic mixture. This procedure was repeated five times. To verify the quality of the ternary eutectic mixture recovered, ^1^H-NMR spectra were generated from the first to fifth cycles. See Supplementary Figure S9 for the ^1^H-NMR experiment.

### 2.10 Scale-up attempt

The ternary eutectic mixture ChCl: *p*TSA: PA (1:1:1.35) was used to explore the properties of the solvent in an attempt to scale-up the reaction. This was performed in three stages, and it was scaled in the following order: ×10, ×100, and ×500. The last one was carried out in a reactor with a total capacity of 5 L.

## 3 Results and discussion

CNC were obtained by a hydrolysis reaction of commercial cellulose using two stochiometric molar ratios of DES-based on ChCl: *p*TSA (DES 1:1 and DES 1:2) and ternary eutectic mixtures ChCl: *p*TSA: PA (see Figure 1B) to evaluate PA as a coadjutant of the acid catalyst hydrolysis of cellulose. The structures of these DES were confirmed using NMR and FTIR techniques and agree with the literature data (Rodriguez Rodriguez et al., 2019; Wang et al., 2022) (See Supplementary Figures S1–S4).

The hydrolysis reaction was carried out by mixing each binary and ternary DES mixture (ChCl: *p*TSA and ChCl: *p*TSA: PA) with cellulose (3% w/w) based on the mass of DES at a temperature of 80 °C for 1–3 h. A typical procedure involves several washings with deionized cold water and centrifugation of the mixture, discarding the supernatant, as explained in the Experimental section. The final dispersion was sonicated (12 min) and then centrifuged. The supernatant (CNC) was finally oven-dried, obtaining CNC. The color of the dispersed CNC was milky white.

Tables 1, 2 summarize the results obtained, yields, and particle size (length and width, nm) for each binary and ternary DES mixture (ChCl: *p*TSA and ChCl: *p*TSA: PA) used as the solvent for the hydrolysis reaction of cellulose. The performance in Exp. 9 was higher because the acidity of this medium allowed the release of nanometric-sized cellulose after sonication. In contrast, in the experiments with 10% phosphoric acid (Exp. 6) or without phosphoric acid (Exp. 3), the media did not present sufficient acidity for this release, so after sonication, the cellulose mass with nanometric size was lower, with the mass of fibers/chains with a high degree of polymerization being higher. The increase in acidity of Exp. 9 meant improved hydrolysis of cellulose to insoluble oligomers, which released CNC after sonication, and this medium presented a suitable acidity, leading to less mass loss from the production of soluble oligomers/glucose in water.

**TABLE 1:** Experimental conditions for hydrolysis of cellulose using DES (1:1) and ternary eutectic mixtures ChCl: *p*TSA: PA (1:1:0.34) and (1:1:1.35) at 80 °C. Yield (%), length, and width average determined by FESEM for the CNC obtained. Each experiment was performed in triplicate.

Exp.	DES	Time (min)	Yield (%)	FESEM (length, nm)	FESEM (width, nm)
1	ChCl: *p*TSA (1:1)	60	26 ± 4	139 ± 32	27 ± 5
2	ChCl: *p*TSA (1:1)	120	30 ± 5	148 ± 47	28 ± 5
3	ChCl: *p*TSA (1:1)	180	39 ± 6	148 ± 40	27 ± 5
4	ChCl: *p*TSA: PA (1:1:0.34)	60	51 ± 5	143 ± 39	25 ± 7
5	ChCl: *p*TSA: PA (1:1:0.34)	120	56 ± 4	198 ± 23	32 ± 6
6	ChCl: *p*TSA: PA (1:1:0.34)	180	67 ± 8	213 ± 29	32 ± 5
7	ChCl: *p*TSA: PA (1:1:1.35)	60	50 ± 8	—	—
8	ChCl: *p*TSA: PA (1:1:1.35)	120	75 ± 5	—	—
9	ChCl: *p*TSA: PA (1:1:1.35)	180	81 ± 6	232 ± 81	27 ± 9

**TABLE 2:** Experimental conditions for hydrolysis of cellulose CFII using DES 1:2 and ternary eutectic mixtures ChCl: *p*TSA: PA (1:2:0.53) and (1:2:2.12) at 80 °C. Yield (%), length, and width average determined by FESEM for the CNC obtained. Each experiment was performed in triplicate.

Exp.	DES	Time (min)	Yield (%)	FESEM (length, nm)	FESEM (width, nm)
10	ChCl: *p*TSA (1:2)	60	0	—	—
11	ChCl: *p*-TSA (1:2)	120	0	—	—
12	ChCl: *p*-TSA (1:2)	180	5 ± 2	—	—
13	ChCl: *p*TSA: PA (1:2:0.53)	60	27 ± 6		
14	ChCl: *p*TSA: PA (1:2:0.53)	120	32 ± 5	—	—
15	ChCl: *p*TSA: PA (1:2:0.53)	180	36 ± 3	229 ± 31	39 ± 7
16	ChCl: *p*TSA: PA (1:2:2.12)	60	36 ± 4	—	—
17	ChCl: *p*TSA: PA (1:2:2.12)	120	55 ± 1	—	—
18	ChCl: *p*TSA: PA (1:2:2.12)	180	61 ± 9	232 ± 26	33 ± 6

Results in Tables 1, 2 show that the yield of CNC obtained depends on the stochiometric value of precise DES, and the yield of CNC in DES 1:1 is close to 30% (see Table 1, exp. 1–3), while when DES 1:2 is the solvent, no CNC is obtained (see Table 2). An interesting effect of PA on the hydrolysis of cellulose was observed, and in Table 1, an increase in the yield of CNC is observed when a ternary deep eutectic mixture is used, following the sequence DES 1:1< ternary DES 1:1:0.34 < ternary DES 1:1:1.35 (39% < 67% < 81, respectively) when the hydrolysis reaction of cellulose is carried out for 3 h. The same effect can be observed in Table 2 for DES 1:2, where the sequence for the increase in the yield of CNC is 0% < 36% < 61% under the same reaction conditions (80 °C, 3 h). Therefore, when PA is added to the ternary DES mixture, the performance of the processes improves concisely. On the other hand, the results show the effect of time on the hydrolysis reaction, and the highest yield of CNC was obtained after 3 h with both binary DES (see Tables 1, 2). It is essential to highlight that longer reaction times were not considered based on other processes reported in the literature for obtaining CNC and getting a moderate energy cost process (Tan et al., 2015; Babicka et al., 2022; Haron et al., 2022).

Furthermore, to compare the acid catalytic abilities of each DES and each ternary eutectic mixture ChCl: *p*TSA: PA used in this work, we have included the results obtained using PA (10 and 40 wt %) in Supplementary Table S1. As can be observed, a very low yield of the nanomaterial is obtained when PA is used as a solvent for the hydrolysis reaction of cellulose (6% and 8%, respectively). These results demonstrate that PA in moderate concentration lacks the acidic environment for the acid catalysis that this type of reaction requires. In this way, we evaluate the effect of sonication of the CNC in the absence of DES, but no CNC resulted in these experimental conditions. A previous report concerning the preparation of CNC by phosphoric acid hydrolysis produced 76% of nanomaterial when filtered using a Whatman No. 1 filter paper. The experimental conditions were 100 °C, PA 10.7 M, and 90 min. The average size of CNC was 316 ± 127 in length and 31 ± 14 in width (Frost and Johan Foster, 2020). However, a two-step mechanical treatment was applied using a blender for 15 min and horn sonication for 15 min.

On the other hand, Hammett acidity parameters (*H*_0_) have been used to determine the efficiency of the catalytic performance of several ionic liquids (ILs) used in acid-catalyzed reactions, such as esterification of alcohols with carboxylic acid or acid anhydrides in the presence of acidic catalysts, where ILs having the highest acidity demonstrated excellent catalytic activity for esterification (Lunagariya et al., 2017). Recently, Contreras *et al.* (2022) (Sanchez et al., 2022) measured the acidity of several ILs and DES using the concepts of donor and acceptor numbers, DNs and ANs, respectively, proposed by Viktor Gutmann (Temp, 1979). Therefore, Hammett acidity parameters (*H*_0_) and the Gutmann number (AN) were determined for binary DES and each ternary eutectic mixture ChCl: *p*TSA: PA as a single component using 4-nitroaniline and ferrocifen as indicators, respectively. The results are shown in Table 3 (the spectrum obtained is shown in Supplementary Figures S5, S6).

**TABLE 3:** Hammett acidity parameters (*H*_0_) and the Gutmann acceptor number (AN) determined for each binary DES, each ternary eutectic mixture ChCl:*p*TSA:PA, and precise PA. Each experiment was performed in triplicate.

Solvent	H_0_	AN
ChCl: *p*TSA (1:1)	1.51	86.0
ChCl: *p*TSA: PA (1:1:0.34)	1.41	90.0
ChCl: *p*TSA: PA (1:1:1.35)	1.31	94.0
ChCl: *p*TSA (1:2)	1.40	91.0
ChCl: *p*TSA: PA (1:2:0.53)	1.20	92.0
ChCl: *p*TSA: PA (1:2:2.12)	1.07	98.0
PA (85%)	0.90	116.0
Sulfuric acid (96%)	0.65*	126.0

**FIGURE 2:**
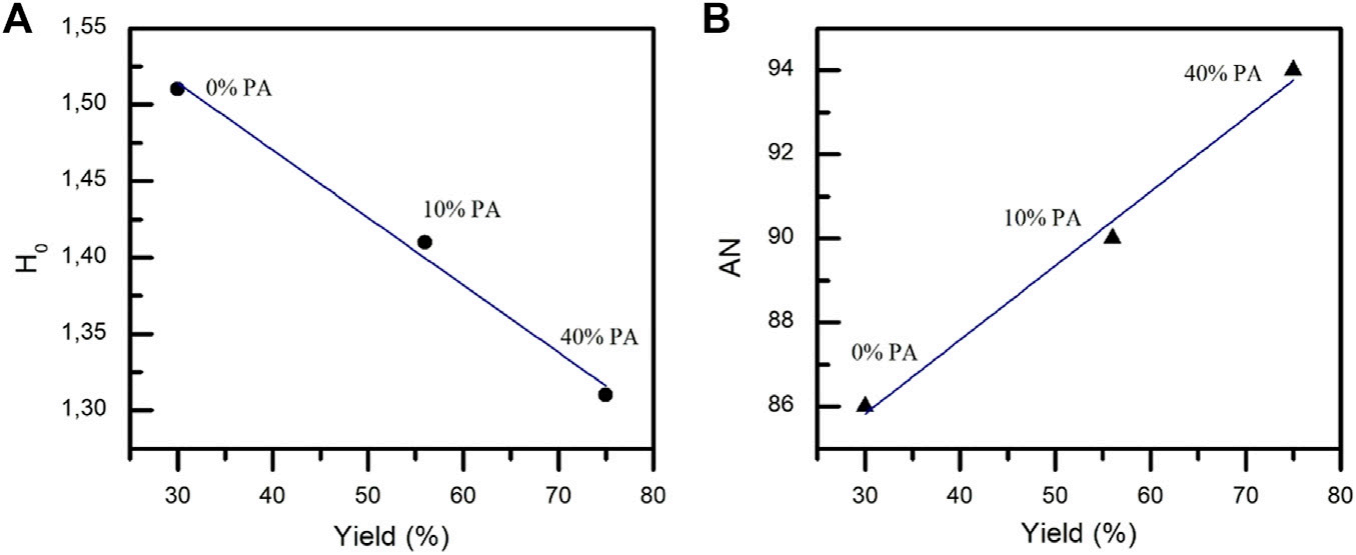
**(A)** Correlation of *H*_0_ values and CNC yields obtained; **(B)** correlation of AN values and CNC yields obtained using DES (1:1), DES (1:1:0.34), and DES (1:1:1.35) as a solvent reaction at 80 °C during 2 h. Each experiment was performed at least in triplicate.

As can be observed in Table 3, the addition of PA affects the acidity of the reaction medium in both cases (DES 1:1 and DES 1:2). As an example, Figure 2 shows a clear linear relationship between both terms of acidity (*H*_0_ and AN values) of DES 1:1, DES 1:1:0.34, and DES 1:1:1.35 and the yield of CNC obtained in each DES when the reaction is performed at 80 °C for 2 h. As can be observed, both acidity parameters indicate a gradual increase of acid capacity catalytic, increasing the reaction yield as the moles of PA in the reaction medium increase. In all experimental conditions used in this study, the behavior is the same. See Supplementary Figure S10.

Moreover, Table 3 shows that the stoichiometry of DES affects both the parameters (*H*_0_ and AN) as well, showing that precise DES 1:2 has a smaller number of *H*_0_ and a higher AN number. This indicates that DES 1:2 provides a much more acidic environment than pure DES 1:1 and, therefore, could explain the low yield of CNC obtained using precise DES 1:2. We have recently reported that the yield of CNC obtained from a hydrolysis reaction using a series of protic ionic liquids based on an anion cluster ([Hmim][(HSO_4_) (H_2_SO_4_)]_x_) as a catalyst and reaction solvent depends on solvent acidity. The results showed that when the IL is a very acidic medium, a low yield of nanomaterial is obtained (Paredes et al., 2022). Nevertheless, when DES ChCl: *p*TSA: PA (1:1:034) is used as a solvent (Exp. 6 Table 1), the yield of CNC improves to 67% despite *H*_0_ and AN numbers being approximately equal to DES ChCl: *p*TSA (1:2) (1.41 and 90 to *H*_0_ and AN parameters, respectively). These results indicate that the cellulose hydrolysis reaction is not only governed by the acidity of the medium. Therefore, factors such as polarity and the ability to donate or accept hydrogen bonds of solvents are parameters that should be considered to understand the effect of the medium on this reaction. Additionally, when DES ChCl: *p*TSA: PA (1:1:1.35) is used as the reaction solvent, the medium acidity increased (1.31 and 94 with *H*_0_ and AN parameters, respectively), and the CNC yield increased to 81%, in accordance with the aforementioned observations. Considering the results of Tables 1, 2, the ternary eutectic mixture ChCl: *p*TSA: PA (1:1:1.35) at 80 °C and 3 h of reaction were the best experimental conditions to obtain 81% yield of CNC.

### 3.1 Morphological analysis of CNC obtained

**FIGURE 3:**
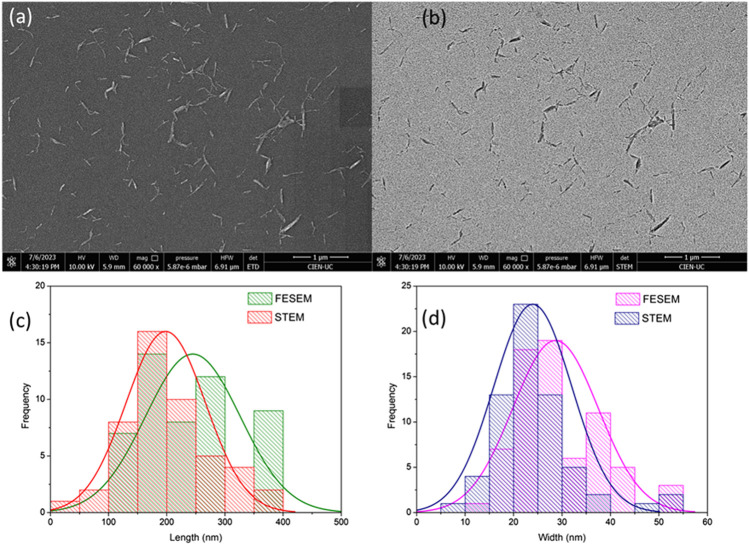
**(A,B)** Comparison between FESEM and STEM techniques, respectively, for determination of the particle size and morphology of CNC obtained using the ternary eutectic mixture DES ChCl: *p*TSA: PA (1:1:1.35) at 80 °C (Exp. 9, Table 1). **(C,D)** Histograms obtained from FESEM and STEM analyses, respectively.

Considering the highest CNC yield obtained in the ternary eutectic mixture DES ChCl: *p*TSA: PA (1:1:1.35), further work was focused on these experimental conditions to obtain CNC. Figure 3 shows a comparison between FESEM and STEM methodologies to determine the particle size and morphology of CNC obtained from the ternary eutectic mixture DES ChCl: *p*TSA: PA (1:1:1.35) at 80 °C. Figure 3A displays FESEM images for nanoparticles of irregular rod-shaped morphology (Paredes et al., 2022). Figure 3B displays STEM images for the same samples where a similar morphology of FESEM is observed. Figures 3C and D show histograms from dimensional analysis of the figures described previously, showing the average length and width of the nanomaterial. As can be observed, there is a correlation between both techniques, where for instance, the higher size frequency in the nanomaterial width ranges from 30 to 45 nm for both techniques, respectively. A similar tendency is observed for the length, where peak lengths of nanocellulose range from 220 to 250 nm for FESEM and STEM, respectively. Therefore, both methodologies yield results in the same order of magnitude. The sizes of CNC obtained in this work are similar to those of CNC extracted by acid hydrolysis with other solvents (Lazko et al., 2016; Yang et al., 2019).

On the other hand, Supplementary Figure S8 shows the FTIR spectra of the different CNCs obtained under different experimental conditions. The results in Supplementary Figure S8 show that the spectra of CNC obtained in experiments 3, 5, 9, and 18 maintain a similar shape to the spectrum of the commercial CFII cellulose, and all bands corresponding to cellulose are observed. See Supplementary Figure S8 for details of the assigned band in FT-IR.

### 3.2 Thermal properties of the obtained CNC

**FIGURE 4:**
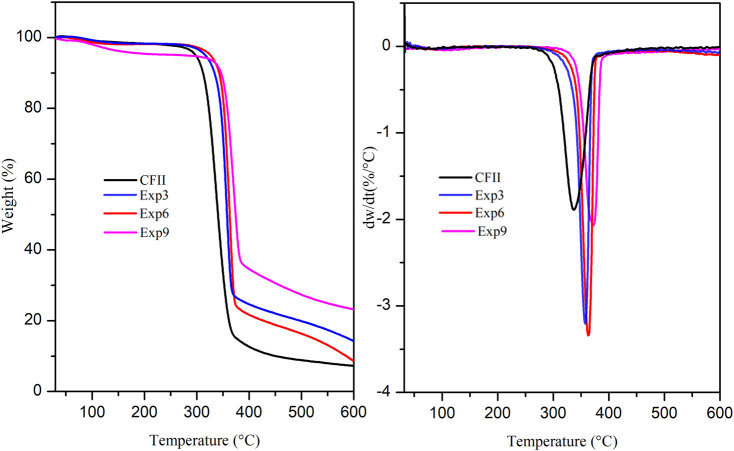
DTG curves for CNC obtained using the binary mixture ChCl: *p*TSA (1:1) and ternary mixtures ChCl: *p*TSA: PA (1:1:0.34) and ChCl: *p*TSA: PA (1:1:1.35) at 80 °C and 3 h. Exps 3, 6, and 9 in Table 1, respectively.

The thermal stability of CNC obtained in the eutectic mixtures ChCl: *p*TSA (1:1), ChCl: *p*TSA: PA (1:1:0.34), and ChCl: *p*TSA: PA (1:1:1.35) (Exps 3, 6, and 9 in Table 1) at 80 °C and 3 h of reaction time was tested using thermogravimetric analysis (TGA), as shown in Figure 4. The results indicate that the addition of PA, to form both ternary eutectic mixtures, increases the thermal stability of the nanomaterial obtained in this experimental condition, as seen in the following sequence. Tmax were 370 ºC, 374 ºC, and 386 °C for ChCl: *p*TSA (1:1), ChCl: *p*TSA: PA (1:1:0.34), and ChCl: *p*TSA: PA (1:1:1.35), respectively. These results are in accordance with the thermogravimetric analyses of CNC obtained with concentrated PA (P-CNC) and those obtained with concentrated sulfuric acid (S-CNC), which show that P-CNC exhibits a much higher thermal stability than S-CNC due to the level of surface functionalization in those S-CNC (Vanderfleet et al., 2018; Frost and Johan Foster, 2020). It is important to note that the amount of phosphate groups on CNC surfaces determines the thermal stability of the CNC produced (Frost and Johan Foster, 2020). Interestingly, our results showed that when we use ChCl: *p*TSA: PA (1:1:1.35) (Exp. 9), the CNC obtained presents the best thermal stability; therefore, the lowest mass loss is observed (see Figure 4). Figure 4 shows that exp. 9 (DES medium with H_2_PO_4_ 40%) presented the highest % residual mass, followed by exp. 6 (DES medium with H_2_PO_4_ 10%) and exp. 3 (DES medium without H_2_PO_4_) with the lowest % residual mass. The sample from exp. 9 is related to better thermal stability (lower value of mass loss rate), and it implies that a higher phosphate content reflected in a higher % residual mass due to C-O cleavage by thermal decomposition of the phosphate ester group on the CNC surface occurred at >500 °C, which involved formation of phosphate salts as solid products along with char. Pristine CNC exhibited a complete loss mass at approximately 500 °C, so phosphorylated CNC produced using the herein ternary system presented a structural advantage acting as a potential flame retardant due to a formation of a protective char layer at above 500 °C (Fiss et al., 2019).

### 3.3 X-ray diffraction analysis

**FIGURE 5:**
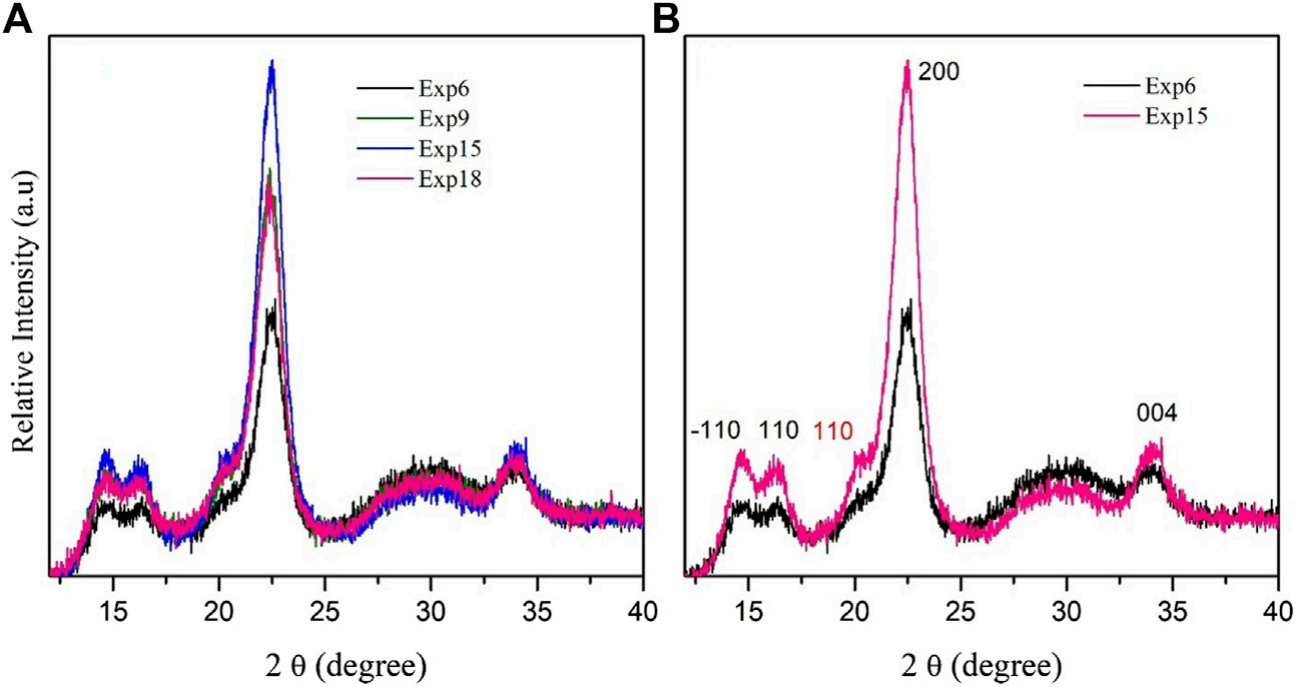
XRD patterns of **(A)** CNC obtained in the experimental condition (Exps 6, 9, 15, and 18 in Tables 1, 2) using the ternary eutectic mixtures ChCl: *p*TSA: PA (1:1:0.34), ChCl: *p*TSA: PA (1:1:1.35), ChCl: *p*TSA: PA (1:2:0.53), and ChCl: *p*TSA: PA (1:2:2.12). **(B)** Comparison of XRD patterns of CNC obtained under the experimental conditions of Exps 6 and 15 (Table 1) using ChCl: *p*TSA: PA (1:1:0.34) and ChCl: *p*TSA: PA (1:1:1.35), respectively.

Figure 5A shows the XRD diffraction patterns of CNC cellulose obtained using the ternary eutectic mixtures ChCl: *p*TSA: PA (1:1:0.34), ChCl: *p*TSA: PA (1:1:1.35), ChCl: *p*TSA: PA (1:2:0.53), and ChCl: *p*TSA: PA (1:2:2.12) at 80 °C (Exps 6, 9, 15, and 18 in Tables 1, 2). All the CNCs obtained using the ternary eutectic mixture ChCl: *p*TSA: PA exhibit similar diffraction peaks and relative intensities. The peaks at 2θ at approximately 14.7° and 16.4° correspond to crystallographic planes overlapping (−110) and (110) peaks characteristic of crystallographic semicrystalline materials. The peaks at 2θ at approximately 22.4° and 34.0° correspond to the (200) and (004) planes characteristic of crystalline cellulose polymorphism Iβ (French, 2014). The peak at 2θ at approximately 20.3° is associated with the (110) lattice plane characteristic of the cellulose II structure. It can be seen in more detail in Figure 5B, comparing XRD diffraction patterns of CNC obtained using ChCl: *p*TSA: PA (1:1:0.34) and ChCl: *p*TSA: PA (1:1:1.35) (Exps 6 and 8 in Table 1, respectively). The results show the absence peak at approximately 20.3° in CNC obtained using ChCl: *p*TSA: PA (1:1:0.34) (Exp. 6, Table 1) indicates a slight change in the conformational crystalline structure of this sample due to the experimental conditions.

In addition, Figure 5B shows broader peaks at approximately 22.4° in the CNC obtained using ChCl: *p*TSA: PA (1:1:1.35) compared with the CNC obtained using ChCl: *p*TSA: PA (1:1:0.34), which can be explained by a smaller crystallite size for the CNC, resulting in this experimental condition. These last results agree with the smaller crystallite size (4.9 nm) obtained using ChCl: *p*TSA: PA (1:1:1.35) compared to the value found for CNC obtained using ChCl: *p*TSA: PA (1:1:0.34) (6.2 nm). See Supplementary Table S3.

**FIGURE 6:**
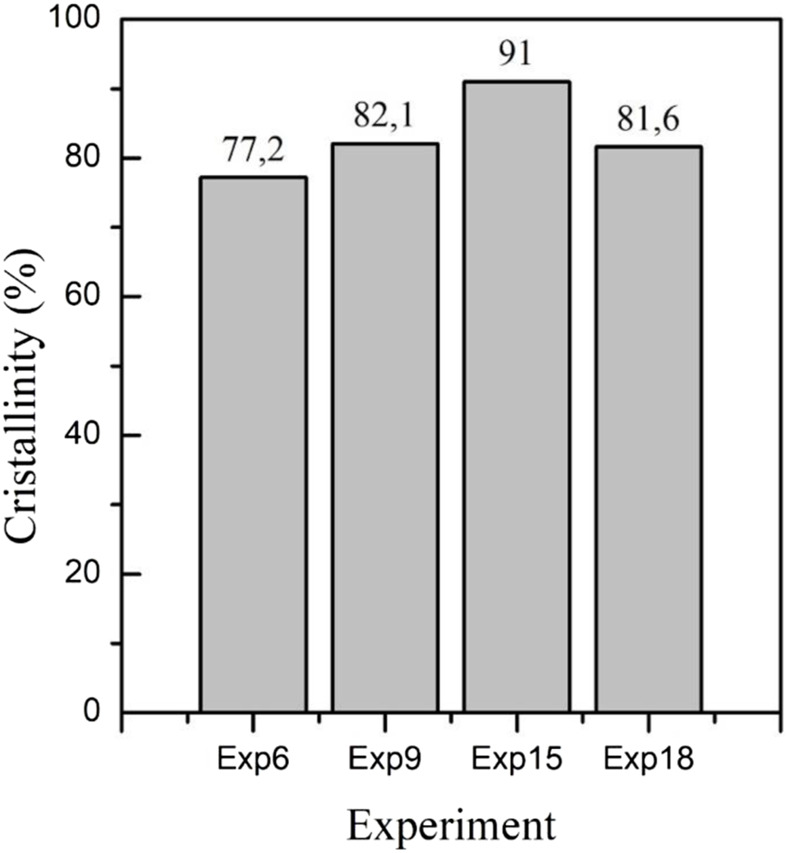
Crystallinity values for CNC obtained using ternary eutectic mixtures ChCl: *p*TSA: PA (1:1:0.34), ChCl: *p*TSA: PA (1:1:1.35), ChCl: *p*TSA: PA (1:2:0.53), and ChCl: *p*TSA: PA (1:2:2.12) by XRD data.

In contrast, the crystallinity index (CrI) of cellulose has been used for a long time with the idea of clarifying changes in the cellulose structure; however, it has been found that CrI varies significantly depending on the choice of the measurement method (Park et al., 2010). For this reason, we performed CrI measurements obtained from the diffraction patterns by the subtraction method (Kang et al., 2018), and the CrIs obtained are shown in Figure 6.

The crystallinity index (CrI) of CNC obtained under different experimental conditions (Exp. 6, 9, 15, and 18 in Tables 1, 2) using the ternary eutectic mixtures ChCl: *p*TSA: PA (1:1:0.34), ChCl: *p*TSA: PA (1:1:1.35), ChCl: *p*TSA: PA (1:2:0.53), and ChCl: *p*TSA: PA (1:2:2.12) indicates that CNC obtained using ChCl: *p*TSA: PA (1:1:1.35) has the higher CrI, and the CNC obtained using ChCl: *p*TSA: PA (1:1:0.34) shows the lower value.

### 3.4 Recyclability of the DES and mixtures

**FIGURE 7:**
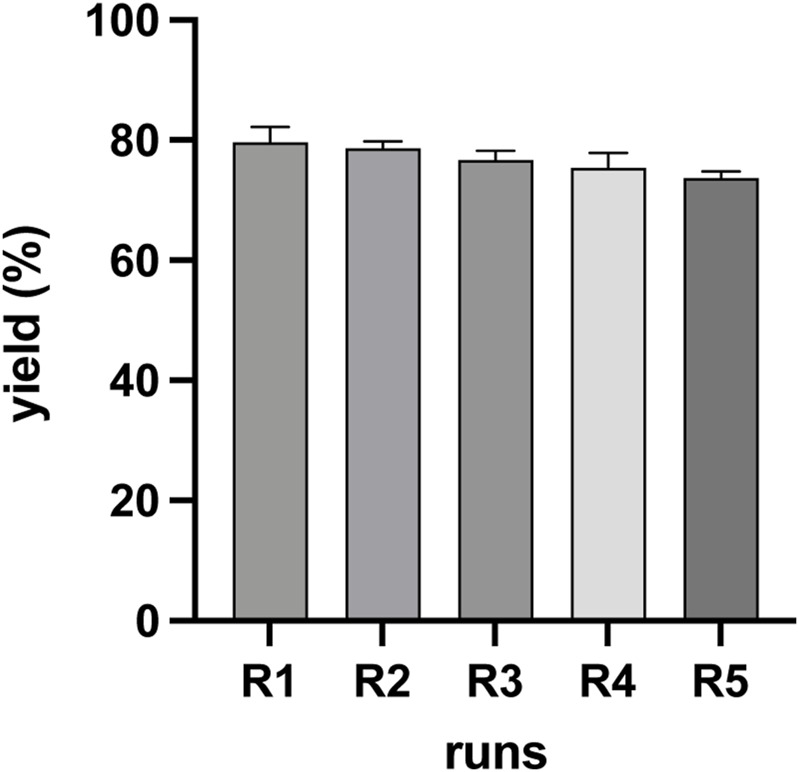
Five cycles of use of DES ChCl: *p*TSA: PA (1:1:1.35) (Exp. 9, Table 1) at 80 °C and 3 h reaction time.

Considering the results in Table 1, the ternary eutectic mixture ChCl: *p*TSA: PA (1:1:1.35) (Exp. 9 in Table 1) was recycled via a reduced pressure distillation method (as explained in the Experimental section) and reused five times to obtain CNC under the same experimental conditions. Figure 7 shows the performance of the recycled mixture to obtain CNC after five cycles. As can be observed, the activity of the recycled mixture has maintained a yield of CNC at approximately 80%, indicating that ChCl: *p*TSA: PA (1:1:1.35) can be reused at least five times as a solvent and catalyst to produce CNC under the experimental conditions. FTIR spectra of the CNC obtained in the last cycle are shown in Supplementary Figure S11. The results show that the morphological features of CNC obtained in each cycle are similar. Additionally, the fresh ChCl: *p*TSA: PA (1:1:1.35) mixture and the recovered mixture after being used five times to obtain CNC were characterized by ^1^H NMR (see Supplementary Figure S9). The results showed no changes in the spectra of the recovered mixture in comparison to the spectra of the fresh ChCl: *p*TSA: PA (1:1:1.35) mixture, indicating that the main chemical compositions of this recovered mixture remain unchanged.

Finally, considering the high yield of CNC obtained (80%) by using the ternary eutectic mixture ChCl: *p*TSA: PA (1:1:1.35) (at 80 °C, 3 h reaction), we attempted to scale the reaction in the same experimental conditions. Considering the mass of ChCl: *p*TSA: PA (1:1:1.35) used in all experiments shown in Table 1, we performed the reaction using mass DESx10, mass DESx100, and mass DESx500 in a reactor (see Supplementary Figure S12). Table 4 shows the yield (%), length, and width average determined by FESEM for the CNC obtained under these last experimental conditions. As can be seen in Table 4, the dimensional and thermal properties of the obtained CNC remain within the same range across all three scales used and are close to those observed in Table 1. However, attempts to scale the reaction to any size reduce the yield to approximately 30%. This is normal for a process in initial optimization. Material losses occur because the operations are more difficult to control during scaling, especially at the thermodynamic level and mass/energy transfer level, and losses are also caused during the flow of fluids between operations. Specifically, to produce NC, there are four operations, reaction, centrifugation, sonication, and centrifugation again, leading to losses in each of these. In addition, viscosity plays a critical role during mass losses by transfer between operations in the case of DES, so dilution with water is an option to consider in an economic analysis to lower costs and reduce viscosity, hence reducing mass losses in each operation (Ma et al., 2019). Nevertheless, the quality of the CNC obtained shows that the heat and mass transfer processes are well-developed. FTIR and thermogravimetric analyses of CNC obtained in all scaling steps are shown in Supplementary Figure S13. The failure in yield could indicate that the final separation, post-chemical reaction, is the process that undoubtedly requires more effort.

**TABLE 4:** Experimental conditions (temperature and time) for hydrolysis of cellulose CFII using ChCl: *p*TSA: PA (1:1:1.35) at the intermediate scale mass DESx10, mass DESx100, and mass DESx500 in a reactor. Yield (%), length, and width average determined by FESEM for the CNC obtained. Each experiment was performed in triplicate.

ChCl: *p*TSA: PA (1:1:1.35)	T (°C)	Time (min)	Yield (%)	FESEM (length, nm)	FESEM (width, nm)	TGA Tonset/Tmax
Mass DESx10	80	180	27 ± 11,7	171 ± 50	35 ± 7	316/332
Mass DESx100	80	180	33 ± 5	154 ± 44	26 ± 6	268/303
Mass DESx500	80	180	36 ± 5	132 ± 50	23 ± 4	339/362

**FIGURE 8:**
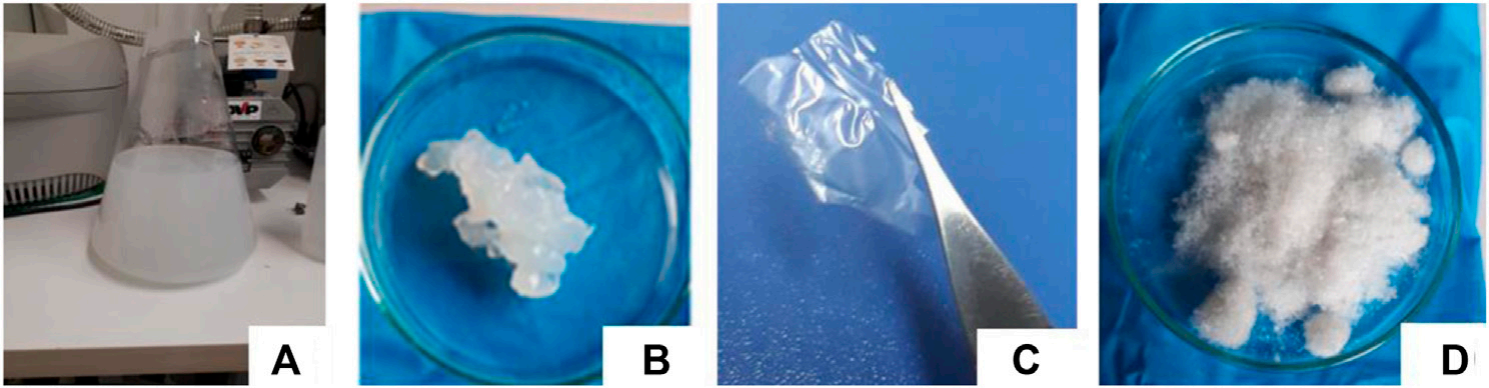
**(A)** CNC suspension; **(B)** CNC gel; **(C)** CNC film; **(D)** CNC powder. All obtained after hydrolysis at 80 °C and 3 h reaction time using the ternary eutectic mixture DES ChCl: *p*TSA: PA (1:1:1.35) (Exp. 9) in the scale mass DESx500 in a reactor.

In addition, Figure 8 shows the different forms of CNC obtained when the reaction was performed in the scaled mass DESx500 in a reactor. As can be seen, it was possible to get (a) CNC suspension water, (b) as a gel (from CNC suspended in water; it is sonicated for 20 min intermittently, with 2-min intervals of sonication, followed by a 1-min pause. Subsequently, it is centrifuged for 15 min at 9,000 rpm. The supernatant is discarded, and the gel is obtained at the bottom of the tube.), (c) as a CNC film (evaporating the water in an oven at 60 °C), and finally, after the lyophilization process, we obtain (d) CNC powder.

## 4 Conclusion

The ternary eutectic mixture ChCl: *p*TSA: PA was evaluated as a hydrolytic system for CNC production from commercial cellulose. The exploration of Hammett acidity function (H_0_) and the Gutmann donor number (AN) elucidated the ternary DES as the suitable acid catalyst system. However, binary and other ternary DES were evaluated for cellulose crystallite release at 80 °C after a mild sonication treatment. Following this process, 3 h of hydrolysis with the ternary DES of molar ratio (1:1:1.35) resulted in the highest yield of 81% at a laboratory scale. Finally, scaling-up of the process was applied in a reactor to obtain a 36% yield of CNC with high thermal stability and an average size of 132 nm × 23 nm. This efficient solvent system is also recyclable at least five times through solid filtration and distillation. A life cycle assessment is necessary to know its potential at the industrial scale.

## Data Availability

The original contributions presented in the study are included in the article/Supplementary Material; further inquiries can be directed to the corresponding author.
