# Supplementary material for: A ternary eutectic solvent for cellulose nanocrystal production: exploring the recyclability and pre-pilot scale-up
Source: Front Chem. 2023 Aug 25;11:1233889. doi: 10.3389/fchem.2023.1233889 (PMC10485260; doi:10.3389/fchem.2023.1233889)
Supplement: Supplementary file 1 [file DataSheet1.docx]

A ternary eutectic solvent for cellulose nanocrystals production: exploring the recyclability and pre-pilot scale-up.

**Mayra A. Mariño^1^, Maria G. Paredes^2^, Natalia Martinez^3^, Daniela Millán^4^, Ricardo A. Tapia^2^, Domingo Ruiz^5^, Mauricio Isaacs^2,6,7*^, Paulina Pavez^2^*.**

^1^ Department of Chemical Engineering, Universidad de Concepción, Concepción, Chile.

^2^ Facultad de Química y de Farmacia, Pontificia Universidad Católica de Chile, Casilla 306, Santiago 6094411, Chile

^3^  Departamento de Química, Universidad Técnica Federico Santa María, Av. Vicuña Mackenna 3939, San Joaquín, Chile

^4^ Universidad Bernardo O’Higgins, Centro Integrativo de Biologia y Quimica Aplicada (CIBQA), General Gana 1702, Santiago, Chile

^5^ Laboratorio de Materiales Electrocerámicos, Facultad de Química y Biología, Universidad de Santiago de Chile, Av. Libertador Bernardo O'Higgins, Santiago, Chile.

^6^ Centro de Nanotecnología y Materiales Avanzados (CIEN-UC), Pontificia Universidad Católica de Chile, Santiago, Chile.

^7^ Millennium Institute on Green Ammonia as Energy Vector. Avda. Vicuña Mackenna 4860. Macul. Santiago Chile.

**Author Information**

* **Correspondence:**

Corresponding author

[**ppavezg@uc.cl**](mailto:ppavezg@uc.cl)

Table of contents

| Content | page |
| --- | --- |
| **Figure S1**. (a) ^1^H-NMR (400 MHz, D_2_0) spectra of ChCl.*p*-toluenesulfonic acid (ChCl:*p-*TSA) DES 1:1 | 3 |
| **Figure S1**. (b) ^1^H-NMR (400 MHz, D_2_0) spectra of ChCl.*p*-toluenesulfonic acid (ChCl:*p-*TSA) DES 1:2 | 3 |
| **Figure S2**. (a) ^13^C-NMR (400 MHz, D_2_0) spectra of ChCl.*p*-toluenesulfonic acid (ChCl:*p-*TSA) DES 1:1 | 4 |
| **Figure S2**. (b) ^13^C-NMR (400 MHz, D_2_0) spectra of ChCl.*p*-toluenesulfonic acid (ChCl:*p-*TSA) DES 1:2 | 4 |
| **Figure S3**. FT**-**IR (pellet in KBr) spectra of ChCl.*p*-toluenesulfonic acid (ChCl:*p-*TSA) DES 1:1 | 5 |
| **Figure S4.** Raman spectra of ChCl.*p*-toluenesulfonic acid (ChCl:*p-*TSA) DES 1:1 | 5 |
| **Figure S5.** Absorbance spectra of 4-nitroaniline after addition of DES ChCl:pTSA (1:1), (1:2) and ternary eutectic mixtures (ChCl:pTSA:PA) in water | 6 |
| **Figure S6.** Uv-visible spectrum of the dye ferrocyphen in DES ChCl:pTSA (1:1), (1:2) and ternary eutectic mixtures (ChCl:pTSA:PA) to determination of Gutmann Number (AN) | 6 |
| **Table S1. ^a^**Experimental conditions for hydrolysis of cellulose CFII and CNC yield obtained using H_3_PO_4_ 10% w/w and **^b^**H_3_PO_4_ 40% w/w with experimental conditions for hydrolysis of cellulose CFII to CNC yield obtained only with 12 min of sonication. | 7 |
| **Figure S7**. (a) FESEM and histograms for CNCs obtained in experimental conditions in Table 1 and Table 2 | 8-10 |
| **Figure S7**. (b) FESEM and histograms for CNCs obtained in recyclable steps | 11 |
| **Figure S7**. (c) FESEM and histograms for CNCs obtained the scale steps | 11 |
| **Table S2.** Thermogravimetric analysis- TGA of CNC obtained using binary eutectic mixture ChCl:*p*TSA (DES1:1) and ChCl:*p*TSA (DES1:2) and ternary eutectic mixtures (ChCl:pTSA:PA) | 11 |
| **Figure S8.** FTIR spectra of CNCs obtained in different experimental conditions. (**-**) Exp.3 ChCl:*p*TSA (1:1); (**-**) Exp. 9 ChCl:*p*TSA:PA (1:1:1.35); (**-**) Exp. 5 ChCl:*p*TSA:PA (1:1:0.34); (**-**) Exp. 18 ChCl:pTSA:PA (1:2:2.12). | 12 |
| **Figure S9.** ^1^H-NMR spectra of ChCl:*p*TSA:PA (1:1:1.35), recycled five times under reaction conditions to obtain CNC, 3h at 80 °C | 13 |
| **Figure S10**. (a) Correlation of H_0_ values and CNC yields (b) correlation of AN values and CNC yields obtained by cellulose hydrolysis by DES (1:1), DES (1:1:0.34), and DES (1:1:1.35) as a solvent reaction at 80ºC during 1 and 3h. | 14 |
| **Table S3.** Crystallinity values of CNC estimated by XRD data. | 14 |
| **Figure S11**. FTIR spectra of the CNC obtained in the last cycle | 14 |
| **Figure S12**. The reactor used in this study (capacity of 5L) | 15 |
| **Figure S13**. (a) FTIR analysis to CNC obtained in all scaling steps | 16 |
| **Figure S13**. (b) Thermogravimetric analysis to CNC obtained in all scaling steps | 17 |


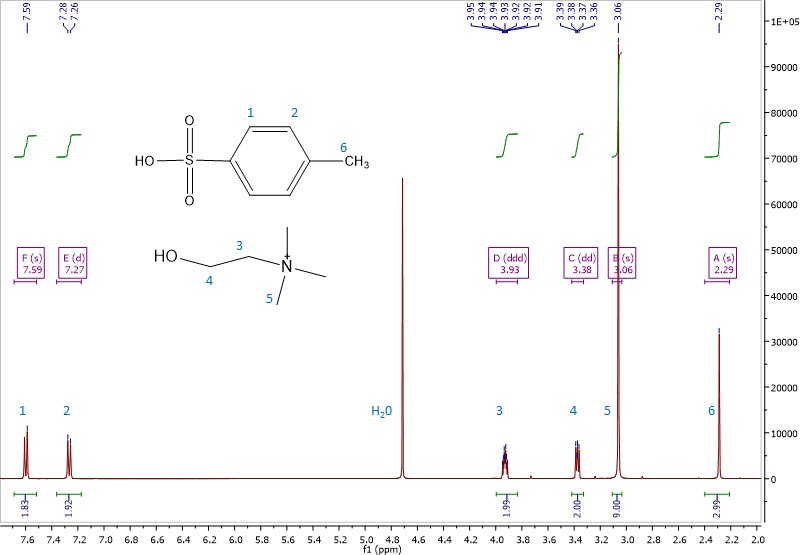


**Figure S1. (a)** ^1^H-NMR (400 MHz, D_2_0) spectra of ChCl.*p*-toluenesulfonic acid (ChCl:*p-*TSA) DES 1:1


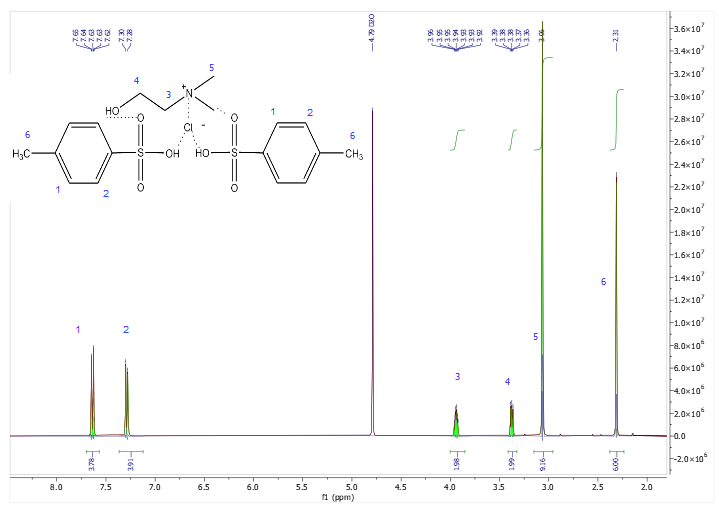


**Figure S1. (b)** ^1^H-NMR (400 MHz, D_2_0) spectra of ChCl.*p*-toluenesulfonic acid (ChCl:*p-*TSA) DES 1:2


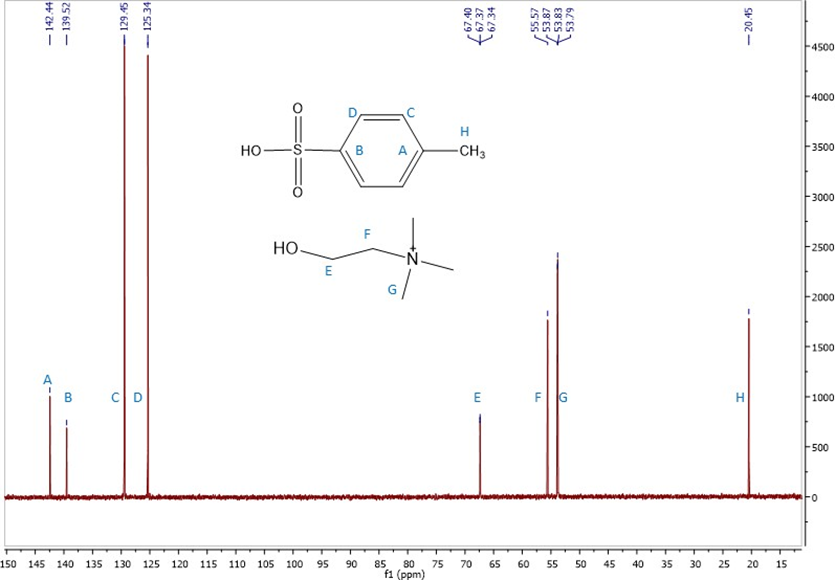


**Figure S2. (a)** ^13^C-NMR (400 MHz, D_2_0) spectra of ChCl.*p*-toluenesulfonic acid (ChCl:*p-*TSA) DES 1:1


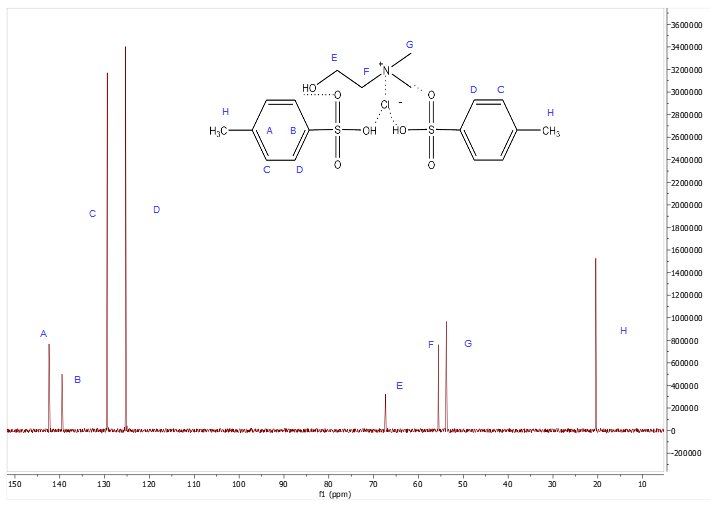


**Figure S2. (b)** ^13^C-NMR (400 MHz, D_2_0) spectra of ChCl.*p*-toluenesulfonic acid (ChCl:*p-*TSA) DES 1:2


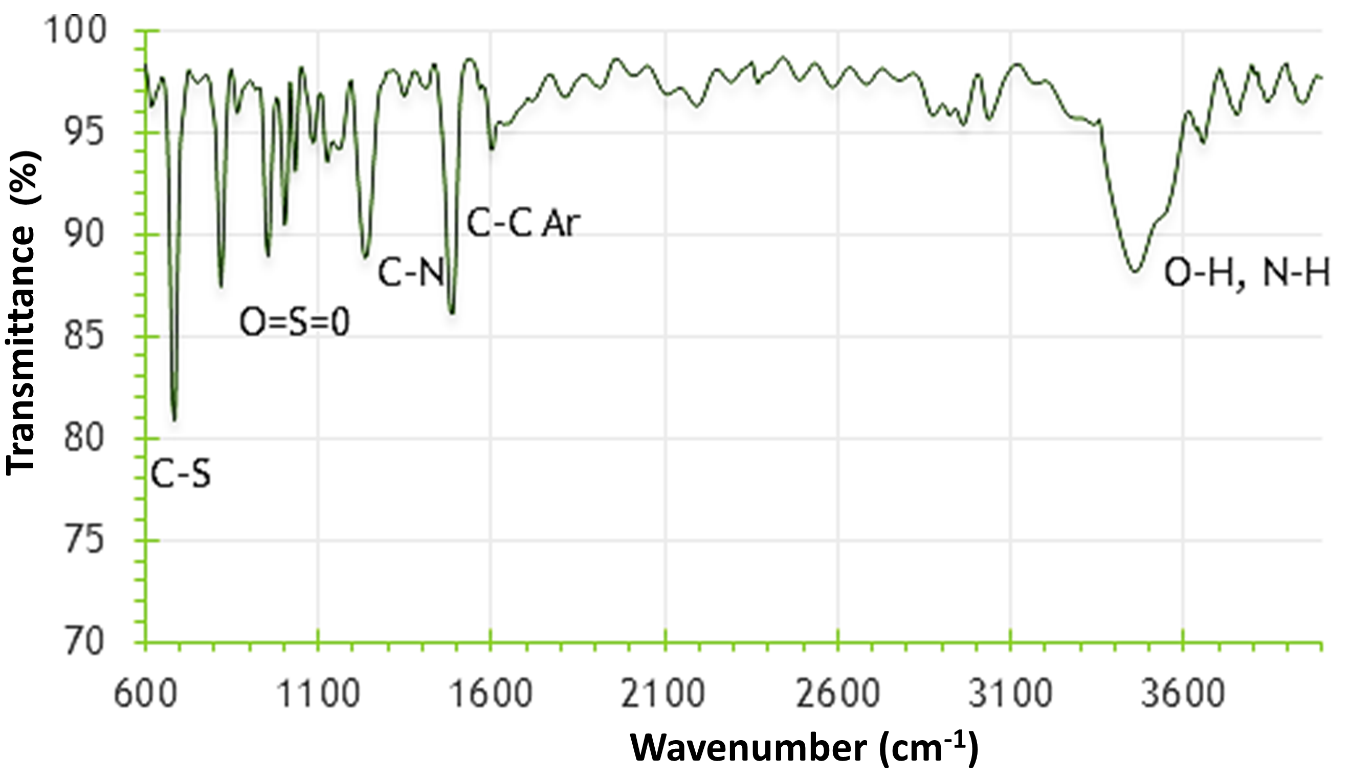


**Figure S3.** FT**-**IR (pellet in KBr) spectra of ChCl.*p*-toluenesulfonic acid (ChCl:*p-*TSA) DES 1:1


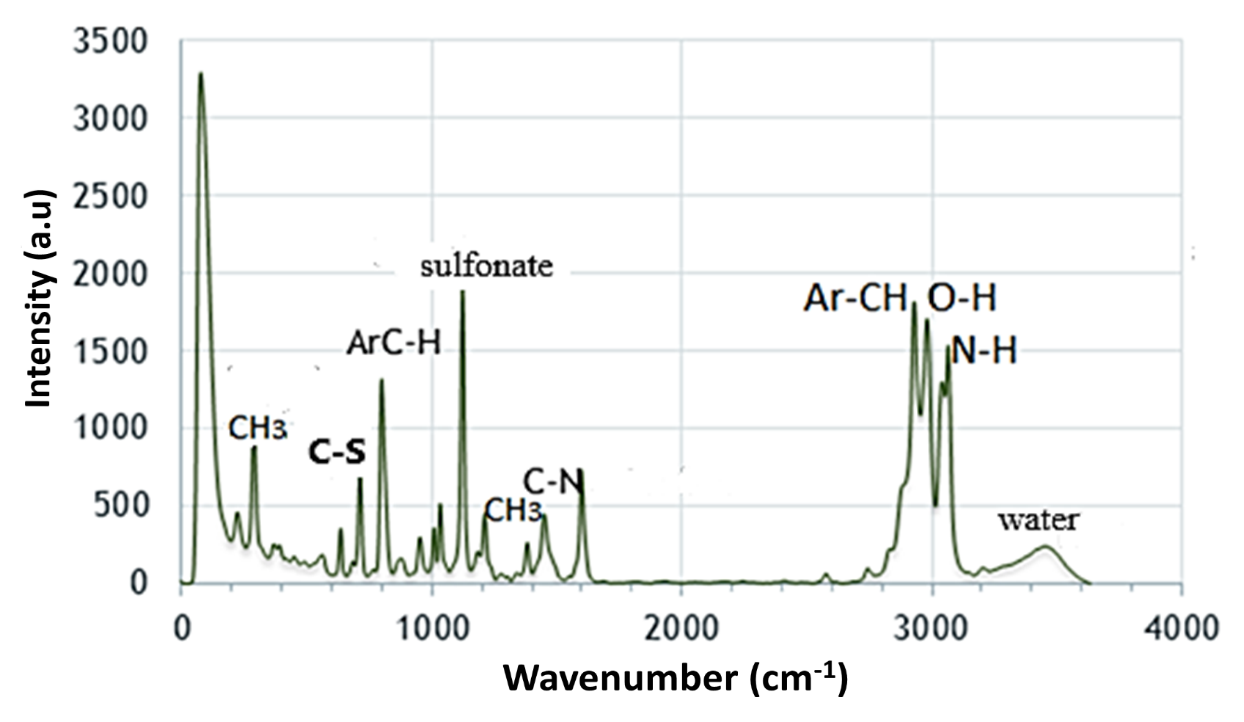


**Figure S4.** Raman spectra of ChCl.*p*-toluenesulfonic acid (ChCl:*p-*TSA) DES 1:1


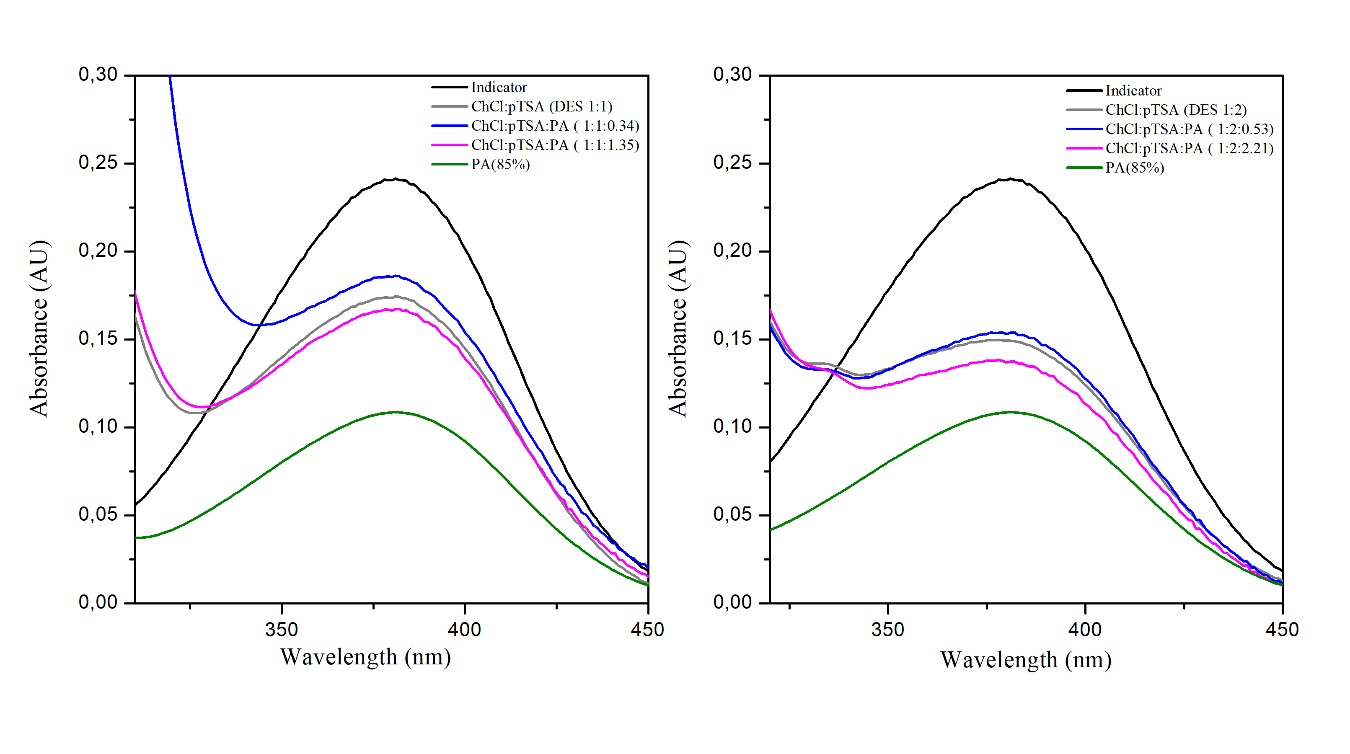


**Figure S5.** Absorbance spectra of 4-nitroaniline after addition of DES ChCl:pTSA (1:1), (1:2) and ternary eutectic mixtures (ChCl:pTSA:PA) in water.


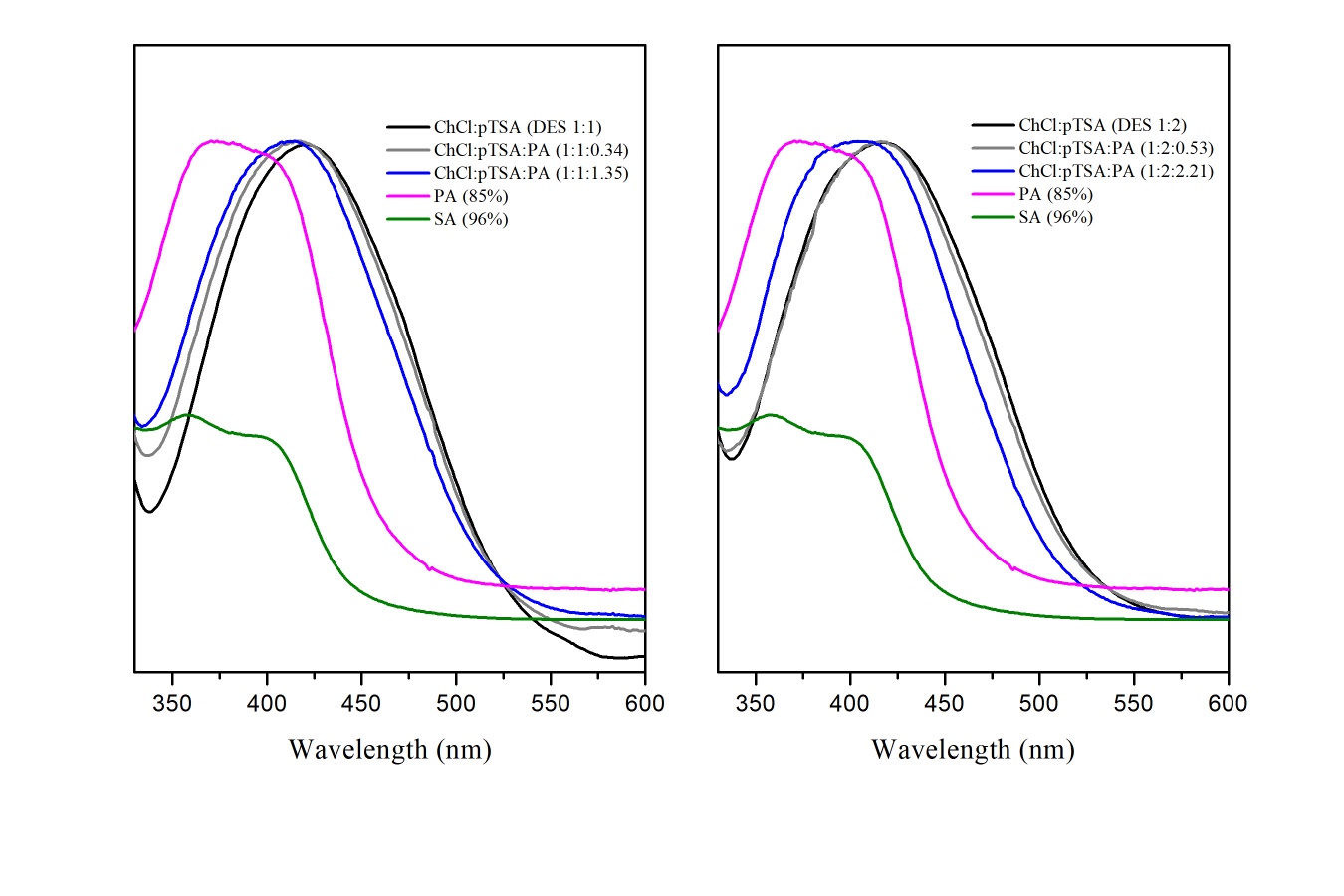


**Figure S6.** Uv-visible spectrum of the dye ferrocyphen in DES ChCl:pTSA (1:1), (1:2) and ternary eutectic mixtures (ChCl:pTSA:PA) to determination of Gutmann Number (AN)

**Table S1. ^a^**Experimental conditions for hydrolysis of cellulose CFII and CNC yield obtained using H_3_PO_4_ 10% w/w and **^b^**H_3_PO_4_ 40% w/w with experimental conditions for hydrolysis of cellulose CFII to CNC yield obtained only with 12 min of sonication

| **Exp.** | **H_3_PO_4_**  **(%)** | **T**  **(°C)** | **time**  **(min)** | **Yield**  **(%)** |
| --- | --- | --- | --- | --- |
| 19**^a^** | 10 | 80 | 180 | 6±5 |
| 20**^b^** | 40 | 80 | 180 | 8±4 |
|  | 0 | - | - | 0 |


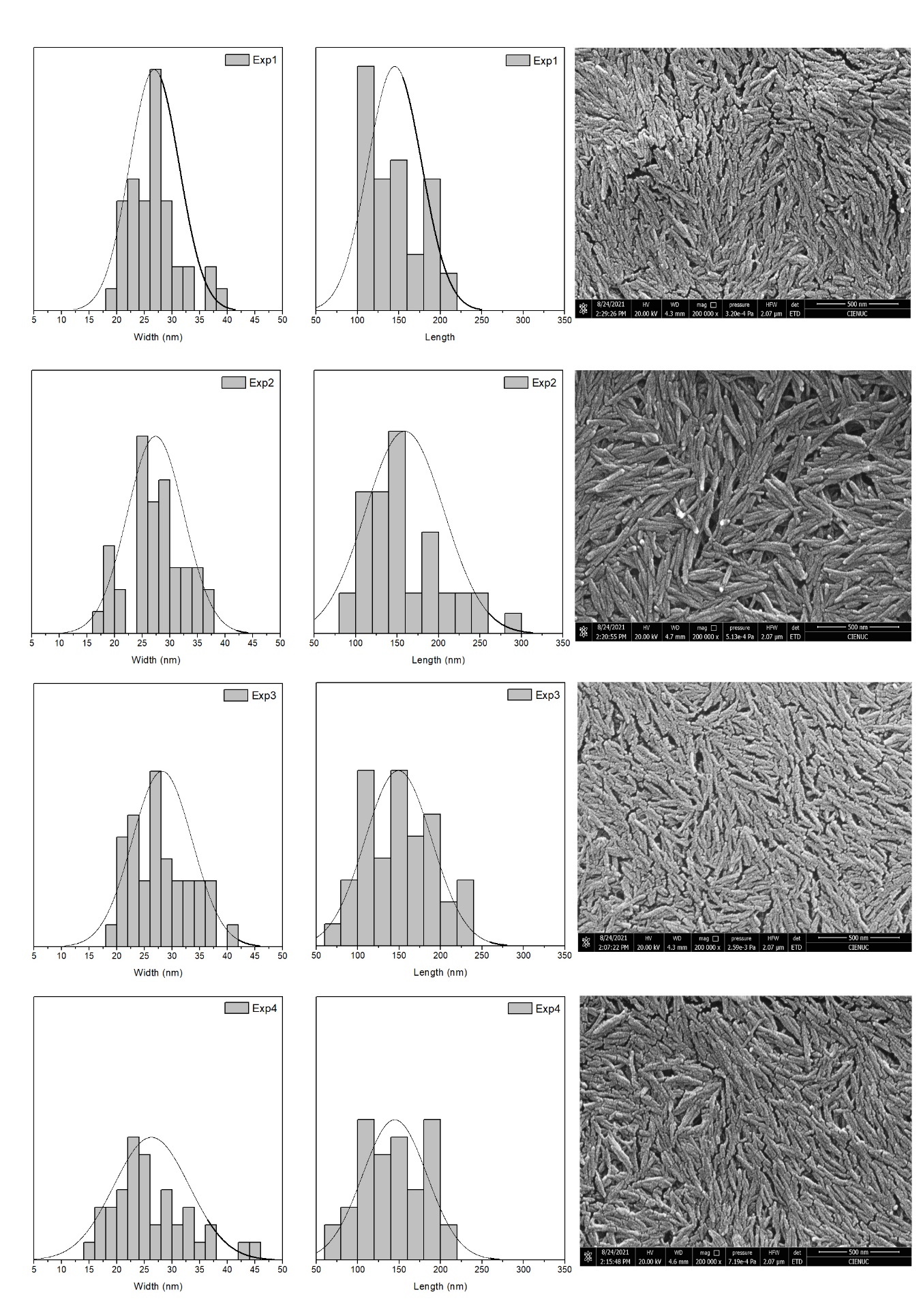


**Figure S7**. (a) FESEM and histograms for CNCs obtained in experimental conditions in Table 1


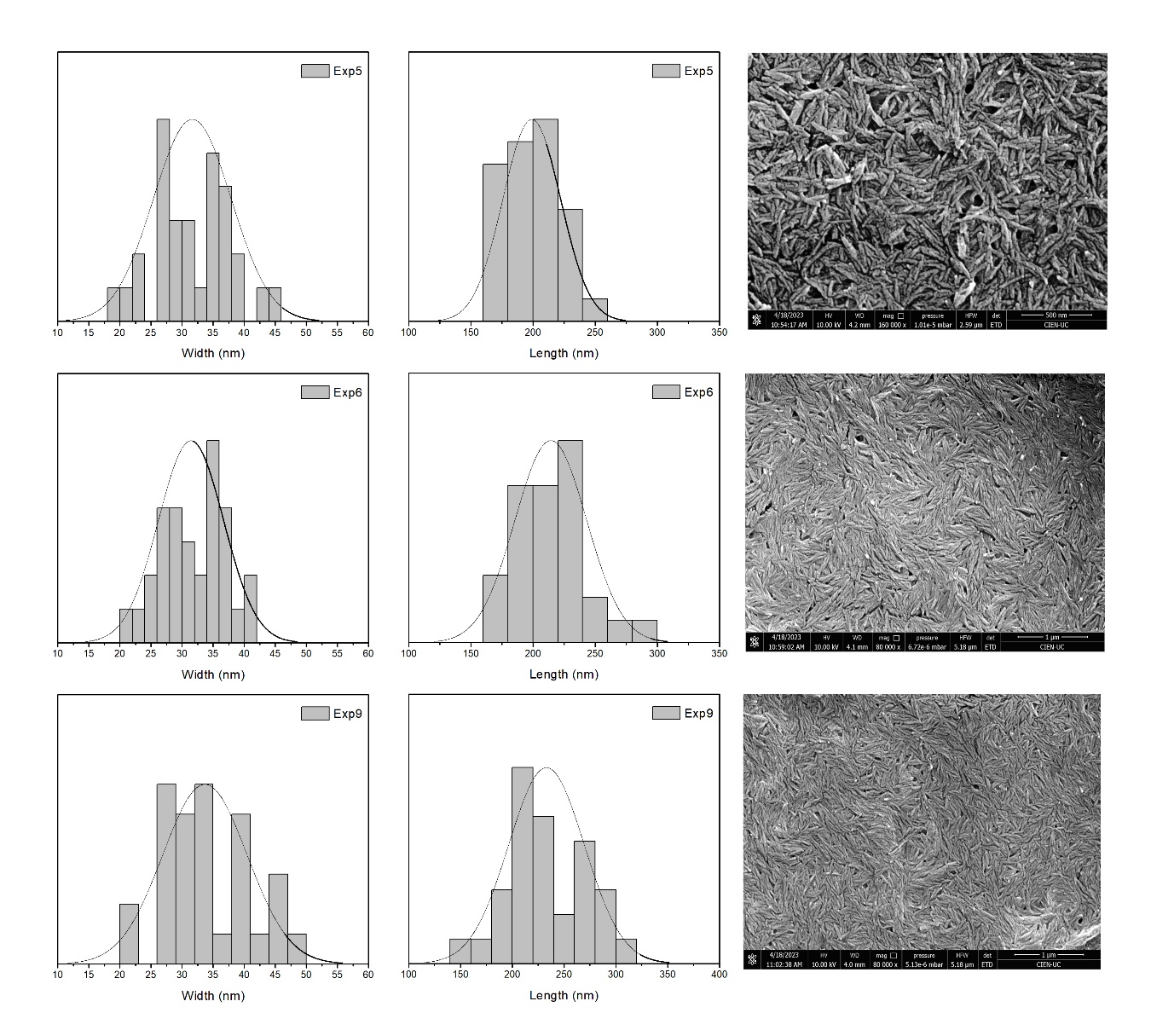


**Figure S7**. (a) FESEM and histograms for CNCs obtained in experimental conditions in Table 1


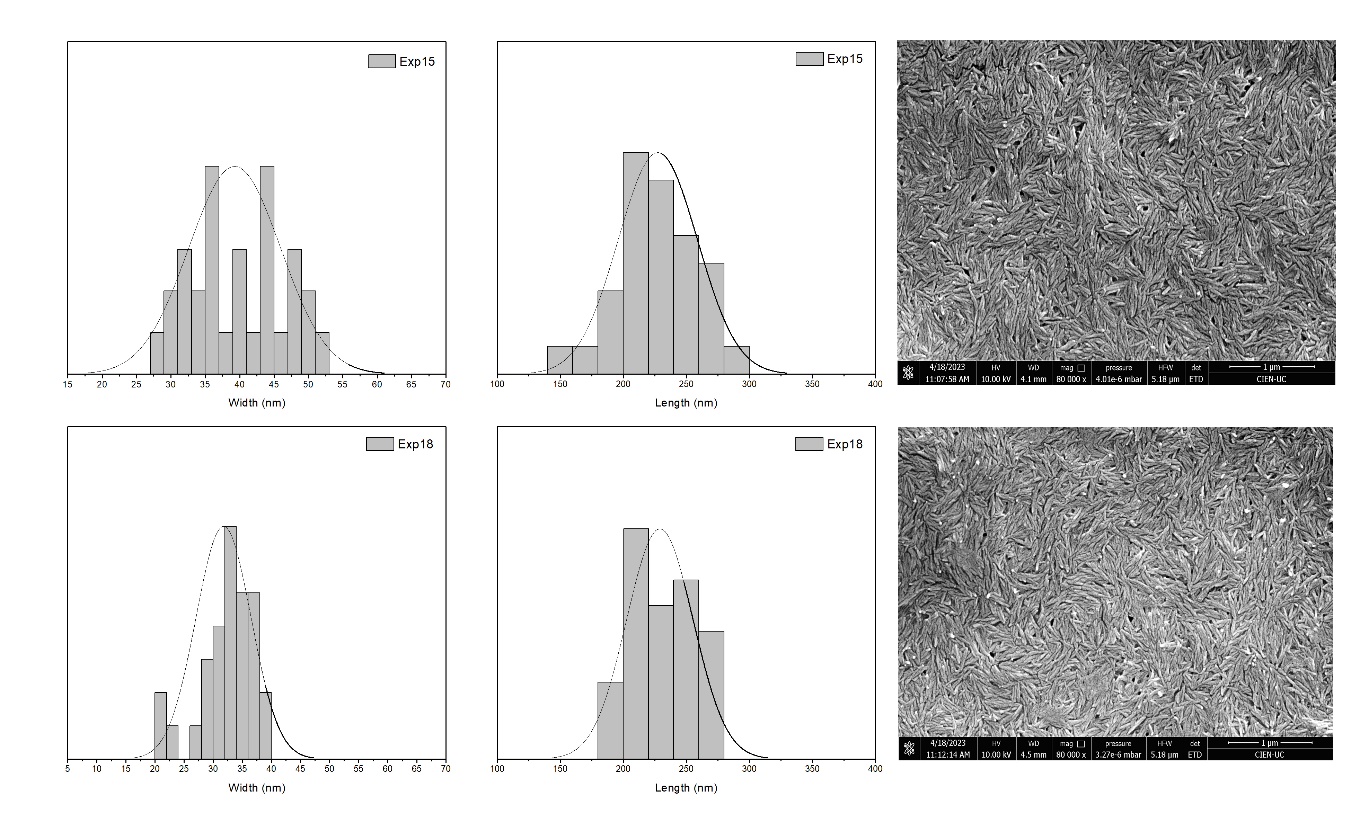


**Figure S7**. (a) FESEM and histograms for CNCs obtained in experimental conditions in Table 2


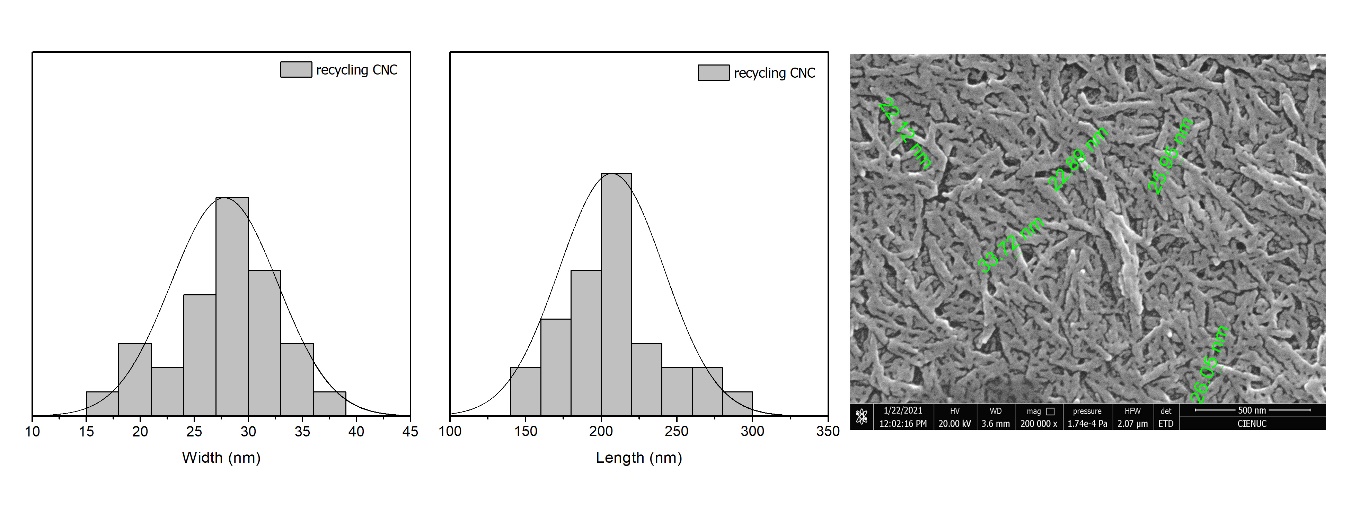


**Figure S7**. (b) FESEM and histograms for CNCs obtained in recyclable steps


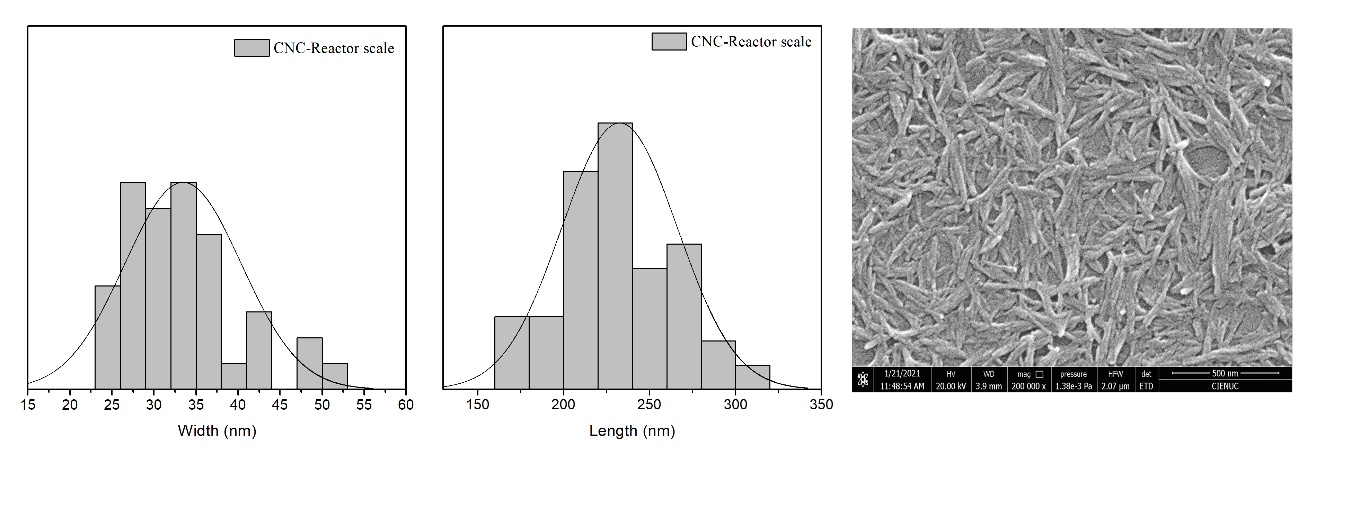


**Figure S7**. (c) FESEM and histograms for CNCs obtained the scale steps

**Table S2.** Thermogravimetric analysis- TGA of CNC obtained using binary eutectic mixture ChCl:*p*TSA (DES1:1) and ChCl:*p*TSA (DES1:2) and ternary eutectic mixtures (ChCl:pTSA:PA)

| **Exp** | **Toneset**  **(°C)** | **Tmax**  **(°C)** |
| --- | --- | --- |
| 1 | 333 | 386 |
| 2 | 327 | 357 |
| 3 | 339 | 370 |
| 4 | 356 | 402 |
| 5 | 349 | 390 |
| 6 | 336 | 374 |
| 9 | 342 | 386 |
| 13 | 336 | 404 |
| 14 | 340 | 380 |
| 15 | 342 | 384 |
| 18 | 366 | 404 |
| CFII | 304 | 367 |





**Figure S8.** FTIR spectra of CNCs obtained in different experimental conditions. (**-**) Exp.3 ChCl:*p*TSA (1:1); (**-**) Exp. 9 ChCl:*p*TSA:PA (1:1:1.35); (**-**) Exp. 5 ChCl:*p*TSA:PA (1:1:0.34); (**-**) Exp. 18 ChCl:pTSA:PA (1:2:2.12)

As we can observe, in all samples a broad band at 3400 cm-1 is observed due to the stretching vibration of the hydroxyl group. The bands around 2900 and 1640 cm-1 are due to C-H stretching vibration and to cellulose-water interactions; respectively. In addition, spectra for all samples show the characteristic bands observed at 1430 cm-1 due to -CH2- (C6) group bending vibration, 1160 due to the stretching C-O-C and, at 895 cm-1 which is associated with C-H rock vibration of cellulose and the characteristic band for the ß-glycosidic linkages between glucose units, see Figure S8. Xing, L.; Gu, J.; Zhang, W.; Tu, D.; Hu, C. Cellulose I and II Nanocrystals Produced by Sulfuric Acid Hydrolysis of Tetra Pak Cellulose I. Carbohydrate Polymers 2018, 192 (February), 184–192. https://doi.org/10.1016/j.carbpol.2018.03.042


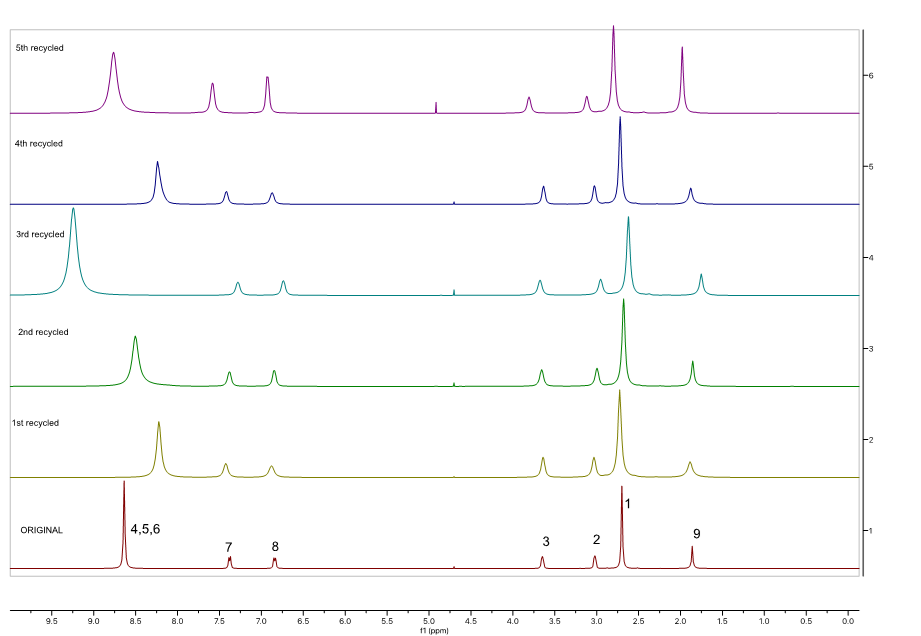


**Figure S9.** ^1^H-NMR spectra of ChCl:*p*TSA:PA (1:1:1.35), recycled five times under reaction conditions to obtain CNC, 3h at 80 °C


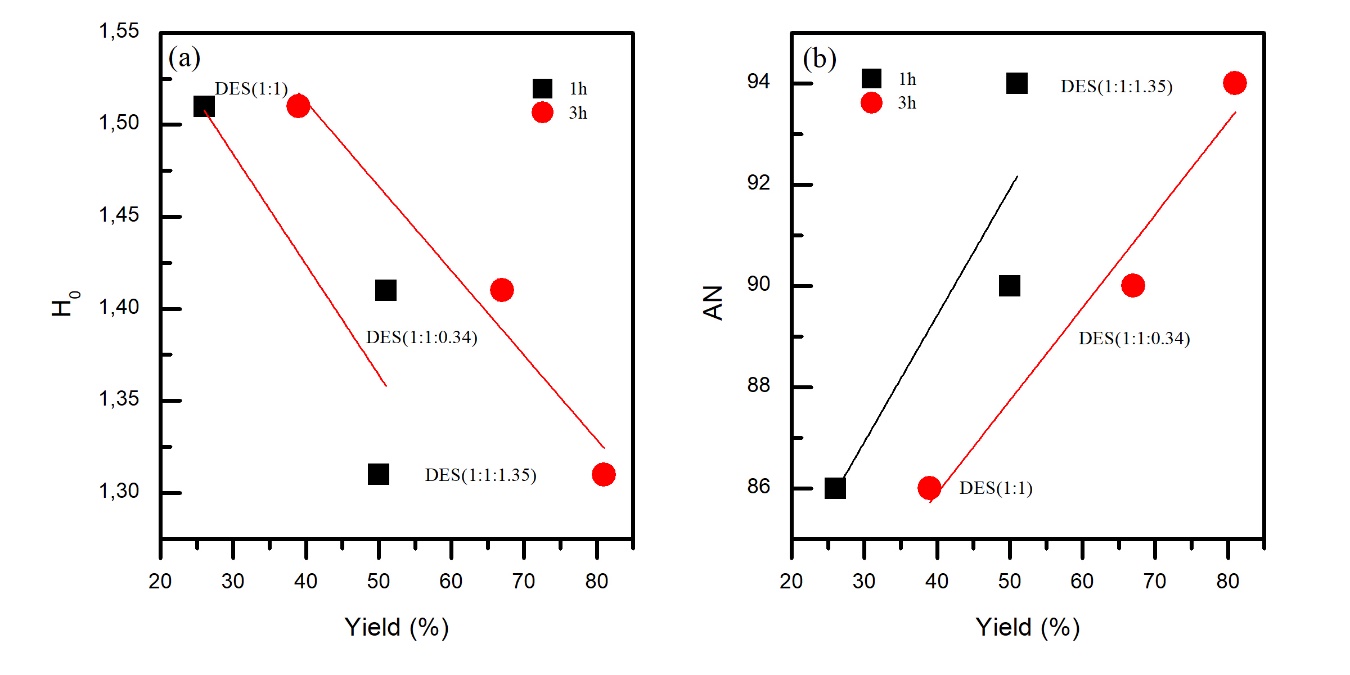


**Figure S10**. (a) Correlation of H_0_ values and CNC yields (b) correlation of AN values and CNC yields obtained by cellulose hydrolysis by DES (1:1), DES (1:1:0.34), and DES (1:1:1.35) as a solvent reaction at 80ºC during 1 and 3h

**Table S3.** Crystallinity values of CNC estimated by XRD data.

| Plane | Crystallite size  (nm) |  |  |  |
| --- | --- | --- | --- | --- |
|  | CNC 6 | CNC 9 | CNC 15 | CNC 18 |
| 200 | 6.2 | 4.9 | 5.9 | 5.9 |


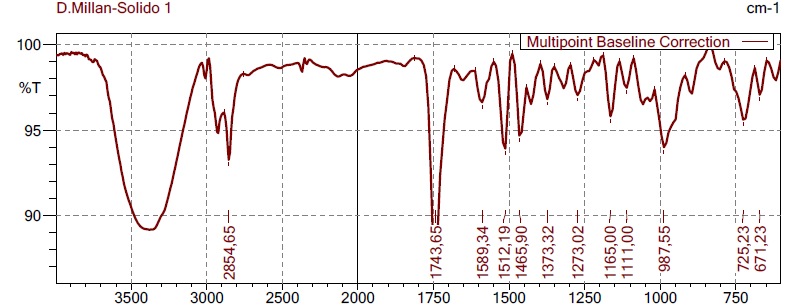


**Figure S11**. FTIR spectra of the CNC obtained in the last hydrolysis cycle


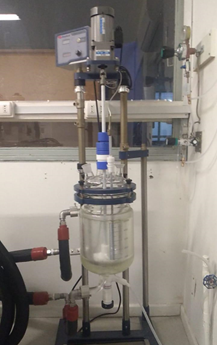


**Figure S12**. The reactor used in this study (capacity of 5L)





**Figure S13**. (a) FTIR analysis to CNC obtained in all scaling steps


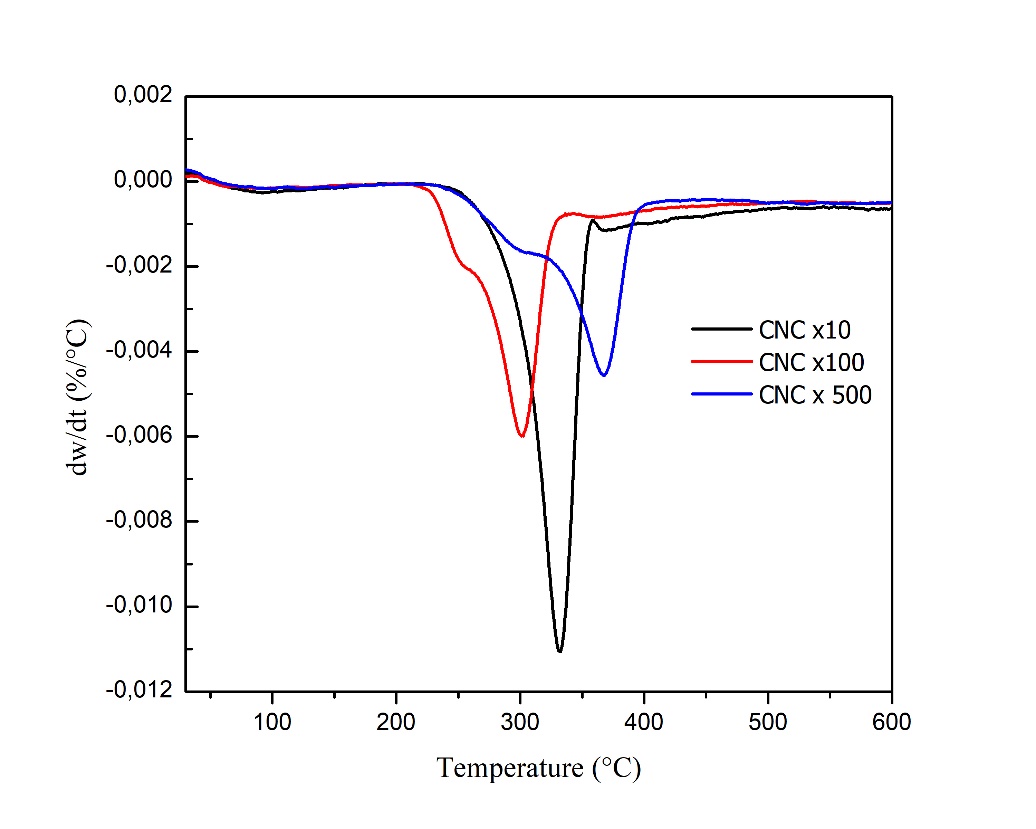


**Figure S13**. (b) Thermogravimetric analysis to CNC obtained in all scaling steps
